# Multi-season transmission model of Eastern Equine Encephalitis

**DOI:** 10.1371/journal.pone.0272130

**Published:** 2022-08-17

**Authors:** Alexa Petrucciani, Geonsik Yu, Mario Ventresca

**Affiliations:** 1 Weldon School of Biomedical Engineering, Purdue University, West Lafayette, Indiana, United States of America; 2 School of Industrial Engineering, Purdue University, West Lafayette, Indiana, United States of America; 3 Purdue Institute for Inflammation, Immunology, and Infectious Diseases, Purdue University, West Lafayette, Indiana, United States of America; University of Texas Medical Branch at Galveston, UNITED STATES

## Abstract

Eastern Equine Encephalitis (EEE) is an arbovirus that, while it has been known to exist since the 1930’s, recently had a spike in cases. This increased prevalence is particularly concerning due to the severity of the disease with 1 in 3 symptomatic patients dying. The cause of this peak is currently unknown but could be due to changes in climate, the virus itself, or host behavior. In this paper we propose a novel multi-season deterministic model of EEE spread and its stochastic counterpart. Models were parameterized using a dataset from the Florida Department of Health with sixteen years of sentinel chicken seroconversion rates. The different roles of the enzootic and bridge mosquito vectors were explored. As expected, enzootic mosquitoes like *Culiseta melanura* were more important for EEE persistence, while bridge vectors were implicated in the disease burden in humans. These models were used to explore hypothetical viral mutations and host behavior changes, including increased infectivity, vertical transmission, and host feeding preferences. Results showed that changes in the enzootic vector transmission increased cases among birds more drastically than equivalent changes in the bridge vector. Additionally, a 5% difference in the bridge vector’s bird feeding preference can increase cumulative dead-end host infections more than 20-fold. Taken together, this suggests changes in many parts of the transmission cycle can augment cases in birds, but the bridge vectors feeding preference acts as a valve limiting the enzootic circulation from its impact on dead-end hosts, such as humans. Our what-if scenario analysis reveals and measures possible threats regarding EEE and relevant environmental changes and hypothetically suggests how to prevent potential damage to public health and the equine economy.

## Introduction

**Fig 1:**
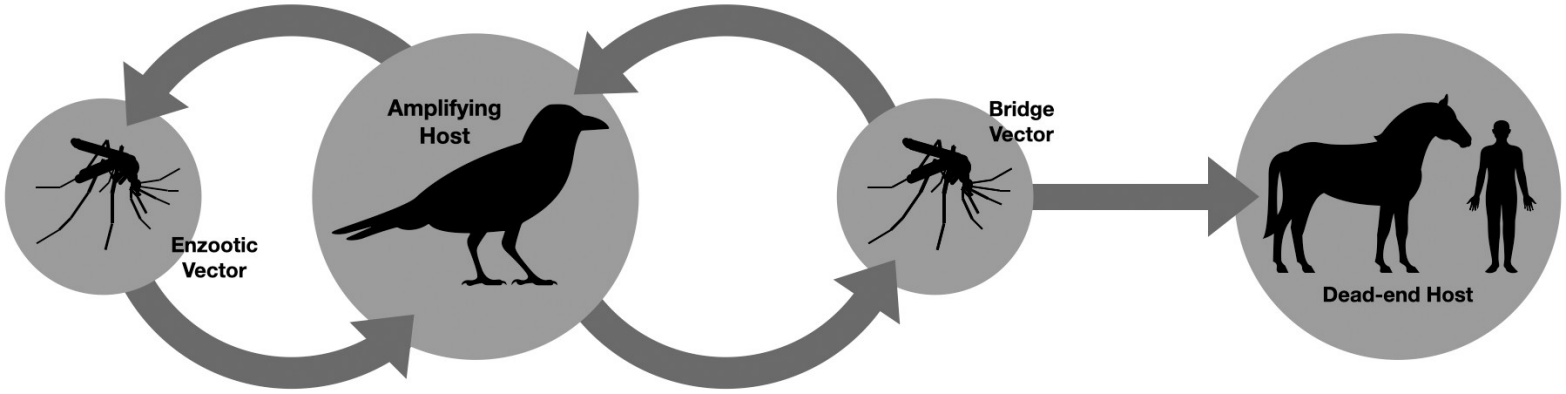
The transmission pathway of EEE. The transmission pathway of EEE consists mainly of a cycle between the enzootic vectors, mainly *Culiseta melanura*, and amplifying hosts, which include many species of birds. Occasionally, transmission through a bridge vector will cause disease in dead-end hosts like humans and horses [2].

Eastern Equine Encephalitis (EEE) is an emerging arbovirus threat. While it was discovered in 1933, cases have remained low in number and relatively limited in geography to eastern and southern coastal areas of the United States [1]. But, in 2019 there was a spike in cases from around 11 yearly to 38 [2]. This peak could be due to changes in climate, weather, human behaviors, or behaviors of other hosts and vectors [3]. EEE generally circulates from spring until fall, and mainly between *Culiseta melanura* mosquitoes and birds, but is occasionally transmitted through a bridge vector to hosts like humans and horses, as shown in Fig 1 [2]. *Ae. albopictus* [4], *Oc. j. japonicus* [5], *Cq. perturbans* [6], and *Cx. erraticus* [7] are known species that can serve as bridge vectors of EEEV.

While the proportion of asymptomatic cases is uncertain, it is estimated that a majority of people who are infected with EEE remain asymptomatic [3]. One small study of the 1959 EEE outbreak in New Jersey suggests less than 5% of those infected develop overt disease of the central nervous system [8]. Of those that are symptomatic there is a 33 percent fatality rate [3], and many of those that survive symptomatic infection have permanent neurological damage. There is no approved human vaccine, preventative medicine, or treatment for EEE, meaning interventions must rely on non-pharmaceutical interventions and supportive treatment. Potential interventions recommended by the CDC include bug spray, wearing long sleeves and pants, and removing stagnant water [2]. These strategies are targeted at decreasing bites and reducing mosquito populations. The deadly nature of the disease, the recent unexplained spike in cases, and the lack of curative care, necessitates a better understanding of the transmission dynamics of EEE and potential consequences if they change and become more dangerous to humans.

While transmission models of other vector-transmitted viruses can be applied to EEE, we are currently aware of only two. One approach focuses on the feeding preferences of *Cs. melanura* on different bird species using an Susceptible-Infected-Removed (SIR) bird and Susceptible-Infected (SI) mosquito model simulated over the course of 180 days [9], finding the Wood Thrush to be an important spreader in Connecticut. The other examines the impact of young-of-the-year (YOY) on transmission over a single season [10]. YOY in this study corresponded to birds younger than 120 days, which are more often successfully fed on by mosquito vectors and more quickly develop higher viral titers. This work suggests that in Alabama YOY are an important driver of EEE transmission. We have yet to find a multi-season and multiyear model of EEE, and we determine that Florida is a good place to base a multi-season model because there is evidence of year round transmission [11], so the question of over-wintering is less confounding. With a multiyear model, yearly variations may be understood better and the impacts of any viral, host, or environmental changes can be explored on a longer time scale.

One concern for the future of EEE is its mutation, especially with the current backdrop of rapid Covid-19 mutations [12–14]. EEE is a Toga virus, which is a family of positive-sense single-stranded RNA viruses that includes Chikungunya and Zika, for instance. RNA viruses, especially +ssRNA viruses, are known to have very high mutation rates, due in part to the error-prone RNA-dependent RNA polymerase that copies their genome [15]. In a recent review of genetic determinants of arboviruses, many Chikungunya mutations were identified [16]. These 4 mutations included those that caused enhanced fitness in 2 separate mosquito species and increased transovarial or vertical transmission. Due to the close relationship between Chikungunya and EEE it is plausible that equivalent mutations could appear in EEE. The genetic variation of EEE isolates suggests that it has a mutation frequency similar to other RNA viruses, although it has a slower observed rate of evolution in nature [17]. This low observed mutation rate is thought to be due to the strong selection pressure from a transmission cycle that requires alternating hosts [18]. However, rapid evolution has been shown to be possible in cell culture [18]. Beyond this, natural variation in EEE virus has been observed, especially over geographically distant isolates [17, 19, 20]. Some of these genetic differences have been associated with changes in virulence [19, 20]. Mutations have also been introduced to EEE with the goal of making attenuated virus for vaccine development [21–24]. These are targeted mutations with the goal of decreasing virulence. Although the rate of evolution is slower in EEE than other RNA viruses, genetic variation and mutations that impact virulence are possible even in nature. Other concerning aspects include the impact of climate change on mosquito populations, the encroachment of humans on wetland regions where EEE has historically circulated, and the possibility of EEE circulating in new mosquito vectors. The numerous possible changes in geographical spread, climate, mosquito behavior, or viral mutations that can impact transmission of EEE along with its deadly, untreatable nature make EEE a potential threat that requires preemptive attention.

In this work, we propose a new multi-season transmission model of EEE, and use this model to explore the impacts of potential viral, host, and environmental changes on the dynamics of EEE spread. After introducing deterministic and stochastic model structures, sentinel chicken data is used to calibrate model parameters. The models are then characterized using *R*_0_, extinction probabilities, and sensitivity analysis. Finally, parameters and models’ structures are adjusted to explore three what-if scenarios and their impacts on EEE transmission.

## Methods

Mathematical models are used to understand the transmission of infectious diseases in populations and to evaluate the potential impact of control programs in reducing morbidity and mortality. We formulate a deterministic model to analyze transmission dynamics and an analogous stochastic epidemic model using a continuous-time Markov Chain. Both models provide actionable information in terms of controlling disease spread and intervention techniques. Employing both models allows us to use a wider variety of analytical techniques because such techniques are designed to work well with one but not with the other. For example, we cannot run a bootstrapping method without having a stochastic model.

### Deterministic model

**Fig 2:**
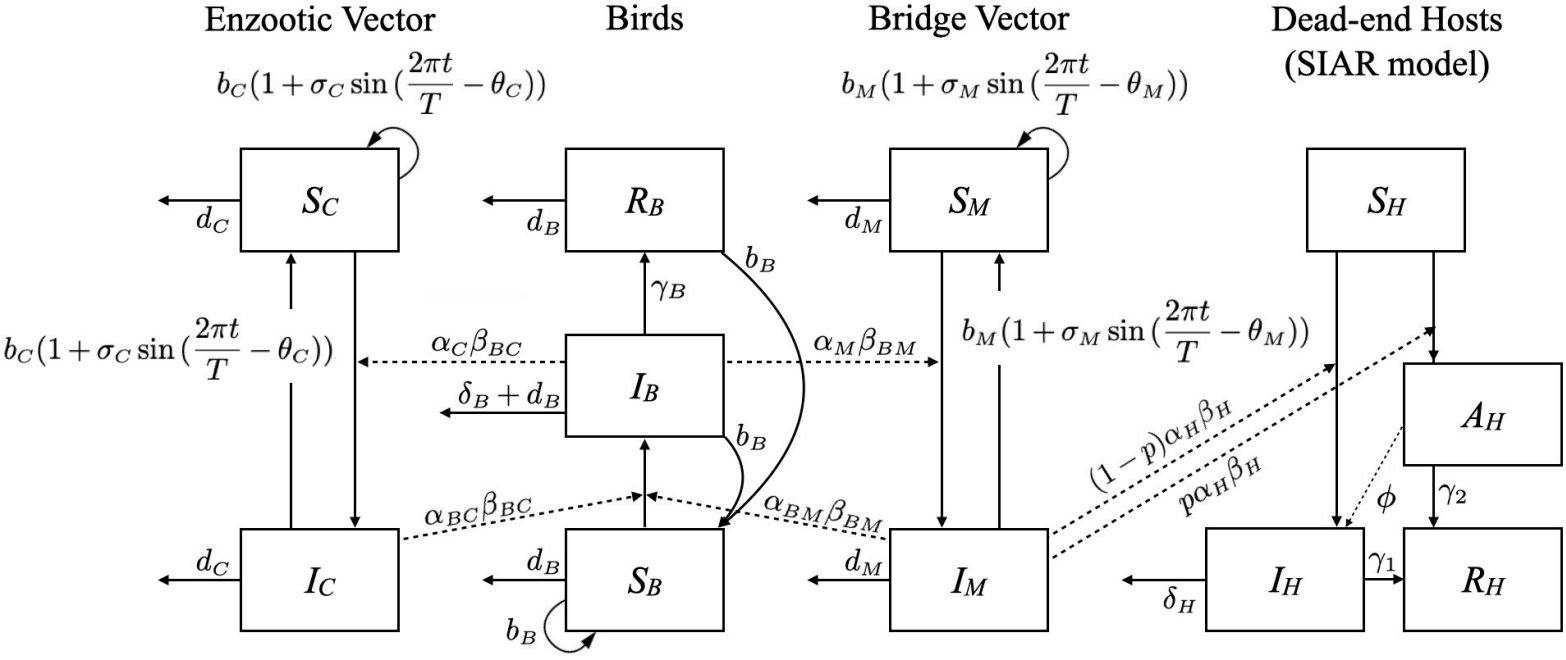
Schematic of the EEE transmission model with seasonal forcing.

A deterministic compartmental model was built on the complex interactions between birds, two types of mosquito vectors, and the dead-end hosts. This transmission pattern as it is currently understood is displayed in Fig 1. The schematic of the model is shown in Fig 2, with parameters as outlined in S2 Appendix.

Although there are different species of mosquitoes with various feeding behaviors, we simplify the model by assuming that there exist only two types (enzootic vector and bridge vector) of mosquitoes with significantly different host preferences. This is because the role of the enzootic vector in circulating EEEV in amplifying host population is important in understanding the disease dynamics of EEEV, whereas the bridge vector is more likely to play the role of infecting dead-end hosts. We assume that bridge vectors mainly feed on mammals and less commonly feed on birds based on the previous studies on host preference of mosquito species [25–27]. Previous studies of blood meal analysis of *Aedes* species show mammal to avian ratios of 83:7 (*Ae. albopictus* [27]), 71:9 (*Ae. triseriatus* [27]), 87:6 (*Ae. vexans* [27]), and 80:11 (*Ae. vexans* [25]). This host preference is reflected by setting *β*_*H*_ = 9 × *β*_*BM*_. This is in contrast to the host preference of major enzootic vector species, *Cx. melanura*, which takes more than 90% of it’s meals from avian sources (97–99% [28], 98.9% [29], 91.3% [30]). Further, we assume that enzootic vectors only feed on birds based on the reported extreme host preference ratios [28–30].

Seasonal forcing was applied to mosquito’s birth rates with a 1-year period, because the onset of the mosquito season is typically when the average temperature reaches 50°*F* and standing water is prevalent. In many locations in the US, this occurs in early June and continues until November. Warmer areas, such as southern Florida, have mosquitoes breeding almost all year round, but there is a marked increase in population size once a year [31]. The magnitude parameters (*σ*_*C*_ and *σ*_*M*_) and the peak location parameters (*θ*_*C*_ and *θ*_*M*_) of seasonal forcing in the two different mosquito groups’ birth rates are assumed independent, following Wilke et al.’s study that suggests species have unique seasonality patterns in Florida. Also, Blosser et al. report that *Culiseta melanura*’s seasonal peak was observed in March and April in Florida in 2015, which is significantly earlier than other species’ peaks in Florida [31, 32]. According to Wilke et al., most of the mosquito species in Florida were most abundant in August or November in 2016, 2017, and 2018. Note that if *σ*_*C*_ = *σ*_*M*_ = 0 the seasonality disappears.

#### Model structure

The ODE system in Eqs (1a)–(1k) shows the proposed deterministic model for EEE.

$$\text{(Enzootic)}\left\{ \begin{array}{cc} \frac{d}{dt}S_{C}=b_{C}\left(1+\sigma_{C}\;\text{sin}\;\left(\frac{2\pi t}{T}-\theta_{C}\right)\right)N_{C}-\alpha_{C}\beta_{BC}\frac{I_{B}}{N_{B}}S_{C}-d_{C}S_{C} & \text{(1a)}\\ \frac{d}{dt}I_{C}=\alpha_{C}\beta_{BC}\frac{I_{B}}{N_{B}}S_{C}-d_{C}I_{C} & \text{(1b)} \end{array}\right.$$

$$\text{(Bridge)}\left\{ \begin{array}{cc} \frac{d}{dt}S_{M}=b_{M}\left(1+\sigma_{M}\;\text{sin}\;\left(\frac{2\pi t}{T}-\theta_{M}\right)\right)N_{M}-\alpha_{M}\beta_{BM}\frac{I_{B}}{N_{B}}S_{M}-d_{M}S_{M} & \text{(1c)}\\ \frac{d}{dt}I_{M}=\alpha_{M}\beta_{BM}\frac{I_{B}}{N_{B}}S_{M}-d_{M}I_{M} & \text{(1d)} \end{array}\right.$$

$$\text{(Birds)}\left\{ \begin{array}{cc} \frac{d}{dt}S_{B}=b_{B}N_{B}-\alpha_{BC}\beta_{BC}\frac{S_{B}}{N_{B}}I_{C}-\alpha_{BM}\beta_{BM}\frac{S_{B}}{N_{B}}I_{M}-d_{B}S_{B} & \text{(1e)}\\ \frac{d}{dt}I_{B}=\alpha_{BC}\beta_{BC}\frac{S_{B}}{N_{B}}I_{C}+\alpha_{BM}\beta_{BM}\frac{S_{B}}{N_{B}}I_{M}-\delta_{B}I_{B}-\gamma_{B}I_{B}-d_{B}I_{B} & \text{(1f)}\\ \frac{d}{dt}R_{B}=\gamma_{B}I_{B}-d_{B}R_{B} & \text{(1g)} \end{array}\right.$$

$$\text{(Hosts)}\left\{ \begin{array}{cc} \frac{d}{dt}S_{H}=-\alpha_{H}\beta_{H}\frac{S_{H}}{N_{H}}I_{M} & \text{(1h)}\\ \frac{d}{dt}A_{H}=\left(1-p\right)\alpha_{H}\beta_{H}\frac{S_{H}}{N_{H}}I_{M}-\phi A_{H}-\gamma_{2}A_{H} & \text{(1i)}\\ \frac{d}{dt}I_{H}=p\alpha_{H}\beta_{H}\frac{S_{H}}{N_{H}}I_{M}+\phi A_{H}-\gamma_{1}I_{H}-\delta_{H}I_{H} & \text{(1j)}\\ \frac{d}{dt}R_{H}=\gamma_{1}I_{H}+\gamma_{2}A_{H} & \text{(1k)} \end{array}\right.$$

For both species of mosquitoes (enzootic and bridge), two compartments were defined, susceptible (*S*_*C*_ or *S*_*M*_) and infectious (*I*_*C*_ or *I*_*M*_), assuming that vectors do not recover from the disease. Birds have 3 compartments susceptible (*S*_*B*_), infectious (*I*_*B*_), and recovered (*R*_*B*_). Births and deaths were incorporated for mosquitoes and birds, but not dead-end hosts, because the lifespans were short relative to the length of simulations. For dead-end hosts, compartments were defined as susceptible (*S*_*H*_), asymptomatic infected (*A*_*H*_), infected with symptoms (*I*_*H*_), and recovered/removed (*R*_*H*_). The meanings, units, and fitted values of all parameters is shown in Table 1 in S2 Appendix. One unit of time variable *t* represents a day accordingly and *T* denotes a year.

### Stochastic model

We also consider a continuous-time Markov chain (CTMC) model for EEE with discrete numbers of hosts and vectors. Stochastic variation in the disease spread model is necessary to consider the randomness caused by different sources such as observation noise, unexpected genotype mutation, and environmental changes. Bartlett pointed out that stochastic fluctuations in disease spread can often be large enough for transmission to be interrupted by stochastic fade-out and deterministic modeling alone cannot explain the disease spread adequately [33]. Especially, as there are currently few cases of EEE in the US that a stochastic approach is better suited. This stochastic model can be examined for small outbreaks that die out quickly, as seen in real-world outbreaks.

#### Model structure

The deterministic model given in Eqs (1a)–(1k) was converted into a continuous-time Markov chain process with transition directions and transition rates shown in Table 1. Eleven discrete-valued random variables are introduced to record the number of individuals in each compartment. For example, *S*_*B*_ is a random variable that tracks the number of susceptible birds over time.

**Table 1:** CTMC transition table. CTMC model transitions between states and rates. For each time interval, the number of each event that occurs is sampled from a Poisson distribution with the mean of *τ* times the corresponding transition rate in the table. When a single event occurs, each compartment’s population changes as described in the ‘Transition’ column.

Species	Event	Transition	Transition Rate
Enzootic Vector	Birth	Δ*S*_*C*_ = 1	$b_{C}\left(1+\sigma_{C}\;\text{sin}\;\left(\frac{2\pi t}{T}-\theta_{C}\right)\right)N_{C}$
	Infection	Δ*S*_*C*_ = −1, Δ*I*_*C*_ = 1	*α*_*C*_*β*_*BC*_(*I*_*B*_/*N*_*B*_)*S*_*C*_
	Death (*S*_*C*_)	Δ*S*_*C*_ = −1	*d*_*C*_ *S*_*C*_
	Death (*I*_*C*_)	Δ*I*_*C*_ = −1	*d*_*C*_ *I*_*C*_
Bridge Vector	Birth	Δ*S*_*M*_ = 1	$b_{M}\left(1+\sigma_{M}\;\text{sin}\;\left(\frac{2\pi t}{T}-\theta_{M}\right)\right)N_{M}$
	Infection	Δ*S*_*M*_ = −1, Δ*I*_*M*_ = 1	*α*_*M*_*β*_*BM*_(*I*_*B*_/*N*_*B*_)*S*_*M*_
	Death (*S*_*M*_)	Δ*S*_*M*_ = −1	*d*_*M*_ *S*_*M*_
	Death (*I*_*M*_)	Δ*I*_*M*_ = −1	*d*_*M*_ *I*_*M*_
Amplifying Host	Birth	Δ*S*_*B*_ = 1	*b*_*B*_ *N*_*B*_
	Infection (from *I*_*C*_)	Δ*S*_*B*_ = −1, Δ*I*_*B*_ = 1	*α*_*BC*_*β*_*BC*_(*S*_*B*_/*N*_*B*_)*I*_*C*_
	Infection (from *I*_*M*_)	Δ*S*_*B*_ = −1, Δ*I*_*B*_ = 1	*α*_*BM*_*β*_*BM*_(*S*_*B*_/*N*_*B*_)*I*_*M*_
	Recovery (*I*_*B*_)	Δ*I*_*B*_ = −1, Δ*R*_*B*_ = 1	*γ*_*B*_
	Death (*S*_*B*_)	Δ*S*_*B*_ = −1	*d*_*B*_ *S*_*B*_
	Death (*I*_*B*_)	Δ*I*_*B*_ = −1	(*d*_*B*_ + *δ*_*B*_)*I*_*B*_
	Death (*R*_*B*_)	Δ*R*_*B*_ = −1	*d*_*B*_ *R*_*B*_
Dead-end Host	Infection (*A*_*H*_)	Δ*S*_*H*_ = −1, Δ*A*_*H*_ = 1	(1 − *p*)*α*_*H*_*β*_*H*_(*S*_*H*_/*N*_*H*_)*I*_*M*_
	Infection (*I*_*H*_)	Δ*S*_*H*_ = −1, Δ*I*_*H*_ = 1	*pα*_*H*_*β*_*H*_(*S*_*H*_/*N*_*H*_)*I*_*M*_
	Transition (*A*_*H*_ → *I*_*H*_)	Δ*A*_*H*_ = −1, Δ*I*_*H*_ = 1	*ϕA*_*H*_
	Recovery (*A*_*H*_)	Δ*A*_*H*_ = −1, Δ*R*_*H*_ = 1	*γ*_2_ *A*_*H*_
	Recovery (*I*_*H*_)	Δ*I*_*H*_ = −1, Δ*R*_*H*_ = 1	*γ*_1_ *I*_*H*_
	Death (*I*_*H*_)	Δ*I*_*H*_ = −1	*δ*_*H*_ *I*_*H*_

### Analytic tools

#### Basic reproduction number

The basic reproduction number (*R*_0_) is the expected number of new infections from one infected individual that is introduced to a wholly susceptible population. This metric is often used as a threshold value for whether an outbreak will occur or not. If *R*_0_ > 1, the disease can cause an outbreak, otherwise, the disease will likely die out. An expression for *R*_0_ can be obtained from the next generation approach [34]. The next-generation matrix (NGM) is a square matrix whose *ij*-th entry is the number of new infections of type *i* from one infected individual of type *j*. Note that there are 5 infectious categories to account for in our model: *I*_*C*_, *I*_*M*_, *I*_*B*_, *A*_*H*_, and *I*_*H*_. The previously described equations for these categories can be broken down into two parts: the new infections (*F*) and the other movement between compartments (*V*). Taking the Jacobian matrices of those two parts at the disease-free equilibrium (DFE) gives us the two components of the NGM. The disease-free equilibrium is [*S*_*C*0_(*t*), 0, *S*_*M*0_(*t*), 0, *S*_*B*0_, 0, 0, *S*_*H*0_, 0, 0, 0] where *S*_*C*0_(*t*) and *S*_*M*0_(*t*) are functions of *t*. That is, *S*_*B*0_ and *S*_*H*0_ are equal to *N*_*B*0_ and *N*_*H*0_, respectively, at the equilibrium.

$$\begin{array}{c} F=[\begin{array}{ccccc} 0 & 0 & \alpha_{C}\beta_{BC}\frac{S_{C0}}{S_{B0}} & 0 & 0\\ 0 & 0 & \alpha_{M}\beta_{BM}\frac{S_{M0}}{S_{B0}} & 0 & 0\\ \alpha_{BC}\beta_{BC} & \alpha_{BM}\beta_{BM} & 0 & 0 & 0\\ 0 & \left(1-p\right)\alpha_{H}\beta_{H} & 0 & 0 & 0\\ 0 & p\alpha_{H}\beta_{H} & 0 & \phi & 0 \end{array}] \end{array}$$ (2)

$$\begin{array}{c} V=[\begin{array}{ccccc} d_{C} & 0 & 0 & 0 & 0\\ 0 & d_{M} & 0 & 0 & 0\\ 0 & 0 & \delta_{B}+d_{B}+\gamma_{B} & 0 & 0\\ 0 & 0 & 0 & \delta_{S}+\gamma_{2} & 0\\ 0 & 0 & 0 & 0 & \gamma_{1}+\delta_{H} \end{array}] \end{array}$$ (3)

The next generation matrix is then calculated as follows:

$$\begin{array}{c} FV^{-1}=[\begin{array}{ccccc} 0 & 0 & \frac{S_{C0}\left(t\right)\alpha_{C}\beta_{BC}}{S_{B0}\left(\delta_{B}+d_{B}+\gamma_{B}\right)} & 0 & 0\\ 0 & 0 & \frac{S_{M0}\left(t\right)\alpha_{M}\beta_{BM}}{S_{B0}\left(\delta_{B}+d_{B}+\gamma_{B}\right)} & 0 & 0\\ \frac{\alpha_{BC}\beta_{BC}}{d_{C}} & \frac{\alpha_{BM}\beta_{BM}}{d_{M}} & 0 & 0 & 0\\ 0 & -\frac{\alpha_{H}\beta_{H}\left(p-1\right)}{d_{M}} & 0 & 0 & 0\\ 0 & \frac{\alpha_{H}\beta_{H}p}{d_{M}} & 0 & \frac{\phi }{\phi +\gamma_{2}} & 0 \end{array}] \end{array}$$ (4)

The spectral radius, or largest eigenvalue, of (4) is the basic reproduction number *R*_0_(*t*). Unlike models without seasonal forcing, the basic reproduction number of our model is a function of *t* and its value depends on when the initial infection occurred. To obtain *R*_0_ at a random time of a year, we integrate *S*_*C*0_(*t*) and *S*_*M*0_(*t*) to compute ${\bar{R}}_{0}$, or the annual *R*_0_ as follows where ${\bar{\nu }}_{c}$ and ${\bar{\nu }}_{m}$ represent the average population ratios between mosquitoes and hosts:

$$\begin{array}{c} {\bar{R}}_{0}=\sqrt{\underset{\text{Transmission}\;\;\text{cycle}\;\;\text{betweenenzootic}\;\;\text{vector}\;\;\text{and}\;\;\text{birds}}{\underset{︸}{\frac{\alpha_{C}\beta_{BC}}{d_{C}}\frac{\alpha_{BC}\beta_{BC}}{d_{B}+\delta_{B}+\gamma_{B}}{\bar{\nu }}_{c}}}+\underset{\text{Transmission}\;\;\text{cycle}\;\;\text{betweenbridge}\;\;\text{vector}\;\;\text{and}\;\;\text{birds}}{\underset{︸}{\frac{\alpha_{M}\beta_{BM}}{d_{M}}\frac{\alpha_{BM}\beta_{BM}}{d_{B}+\delta_{B}+\gamma_{B}}{\bar{\nu }}_{m}}}} \end{array}$$ (5)

where ${\bar{\nu }}_{c}=\frac{\int_{0}^{T}\;S_{C0}\left(t\right)\;dt}{TS_{B0}}$ and ${\bar{\nu }}_{m}=\frac{\int_{0}^{T}\;S_{M0}\left(t\right)\;dt}{TS_{B0}}$.

$$\begin{array}{c} S_{C0}\left(t\right)/S_{B0}=c_{C}\;\text{exp}\;(\left(b_{C}-d_{C}\right)t-\frac{b_{C}\sigma_{C}T\;\text{cos}\;\left(\frac{2\pi t}{T}-\theta_{C}\right)}{2\pi }) \end{array}$$ (6)

$$\begin{array}{c} S_{M0}\left(t\right)/S_{B0}=c_{M}\;\text{exp}\;(\left(b_{M}-d_{M}\right)t-\frac{b_{M}\sigma_{M}T\;\text{cos}\;\left(\frac{2\pi t}{T}-\theta_{M}\right)}{2\pi }) \end{array}$$ (7)

*S*_*C*0_(*t*) and *S*_*M*0_(*t*) can be obtained by solving the following differential equations.

$$\begin{array}{c} \frac{d}{dt}S_{C0}\left(t\right)=b_{c}\left(1+\sigma_{C}\;\text{sin}\;(\frac{2\pi t}{T}-\theta_{C})\right)S_{C0}\left(t\right)-d_{c}S_{C0}\left(t\right) \end{array}$$ (8)

$$\begin{array}{c} \frac{d}{dt}S_{M0}\left(t\right)=b_{m}\left(1+\sigma_{M}\;\text{sin}\;(\frac{2\pi t}{T}-\theta_{M})\right)S_{M0}\left(t\right)-d_{m}S_{M0}\left(t\right) \end{array}$$ (9)

The idea of integrating the seasonal effect terms is originally suggested by Grassly and Fraser [35]. Their study demonstrated that the basic reproduction number does not apply when there exists seasonal forcing in diseases dynamics, and propose the average number ${\bar{R}}_{0}$ at a random time of the year as an alternative.

Eq (5) is composed of two parts that represent the transmission cycle of enzootic vector and bridge vector, respectively. Each part is a multiplication of three fractional terms: (a) mosquito to bird transmission (the number of newly infected mosquitoes per one infected bird divided by mosquitoes’ removal rate from the infectious compartment: $\frac{\alpha_{C}\beta_{BC}}{d_{C}}$ and $\frac{\alpha_{M}\beta_{BM}}{d_{M}}$), (b) bird to mosquito transmission (the number of newly infected birds per one infected mosquito divided by birds’ removal rate from the infectious compartment: $\frac{\alpha_{BC}\beta_{BC}}{\delta_{B}+d_{B}+\gamma_{B}}$ and $\frac{\alpha_{BM}\beta_{BM}}{\delta_{B}+d_{B}+\gamma_{B}}$), and (c) initial mosquito-to-bird ratios ($\frac{\int_{0}^{T}\;S_{C0}\left(t\right)\;dt}{TS_{B0}}$ and $\frac{\int_{0}^{T}\;S_{M0}\left(t\right)\;dt}{TS_{B0}}$). The annual mosquito-to-bird ratios are typically assumed to be greater than 1 in the existing studies [36]. Therefore, if ${\bar{R}}_{0}<1$, then (a) × (b) < 0.5/(*c*), if the parameter sets for the two different vector species are equivalent. As we can see from the Eq (5), dead-end host parameters and population size are not considered in ${\bar{R}}_{0}$ computation. That is, dead-end host’s infection does not affect the disease persistence.

#### Sensitivity analysis

A common importance measure for factors in deterministic models is the elasticity index (or normalized sensitivity index), which measures the relative change of *R*_0_ with respect to a certain factor *x*. In this study, we used $\bar{R_{0}}$ instead of *R*_0_ to compute each factor’s elasticity index to calculate the expected importance of each factor over a year as follows:

$$\begin{array}{c} e_{x}^{\bar{R_{0}}}=\frac{\partial \bar{R_{0}}}{\partial x}\times \frac{x}{\bar{R_{0}}} \end{array}$$ (10)

#### Extinction probability

A Galton-Watson branching process approximation was used to calculate the extinction probabilities of the stochastic model of Table 1 [37–40]. The infectious categories are *I*_*C*_, *I*_*M*_, *I*_*B*_, *A*_*H*_, and *I*_*H*_. The first step is to find the offspring probability-generating functions (PGFs) for each of these 5 categories, assuming that we are near disease-free equilibrium. Offspring PGFs will take the following form:

$$\begin{array}{c} f_{i}\left(u\right)=\Sigma_{k_{n}}...\Sigma_{k_{1}}P_{i}\left(k_{1},...,k_{n}\right)u_{1}^{k_{1}}...u_{n}^{k_{n}} \end{array}$$ (11)

Note that *P*_*i*_(*k*_1_, …, *k*_*n*_) refers to the probability of a type *i* individual producing an individual of type *k*_*j*_. For each probability generating function of type *i*, we assume one infectious individual of type *i* and none of the others. Based on Table 1, we can establish the following PGFs:

$$\begin{array}{c} f_{1}\left(u\right)=\frac{d_{C}+\alpha_{BC}\beta_{BC}u_{1}u_{3}}{d_{C}+\alpha_{BC}\beta_{BC}} \end{array}$$ (12a)

$$\begin{array}{c} f_{2}\left(u\right)=\frac{d_{M}+\alpha_{BM}\beta_{BM}u_{2}u_{3}+\left(1-p\right)\alpha_{H}\beta_{H}u_{2}u_{4}+p\alpha_{H}\beta_{H}u_{2}u_{5}}{d_{M}+\alpha_{BM}\beta_{BM}+\left(1-p\right)\alpha_{H}\beta_{H}+p\alpha_{H}\beta_{H}} \end{array}$$ (12b)

$$\begin{array}{c} f_{3}\left(u\right)=\frac{\alpha_{C}\beta_{BC}u_{1}u_{3}+\alpha_{M}\beta_{BM}u_{2}u_{3}+\left(\delta_{B}+d_{B}+\gamma_{B}\right)}{\alpha_{C}\beta_{BC}+\alpha_{M}\beta_{BM}+\left(\delta_{B}+d_{B}+\gamma_{B}\right)} \end{array}$$ (12c)

$$\begin{array}{c} f_{4}\left(u\right)=\frac{\phi u_{5}+\gamma_{2}}{\phi +\gamma_{2}} \end{array}$$ (12d)

$$\begin{array}{c} f_{5}\left(u\right)=\frac{\gamma_{1}+\delta_{H}}{\gamma_{1}+\delta_{H}}=1 \end{array}$$ (12e)

Note that *u*_*i*_ refers to the corresponding infectious compartment. For example, *u*_1_ corresponds to the number of infected enzootic vectors; *u*_2_, *u*_3_, *u*_4_, *u*_5_ refer to the infected bridge vectors, infected birds, asymptomatic humans, and symptomatic humans, respectively.

Equations *f*_*i*_ have 3 fixed points in [0, 1]^5^, specifically [1, 1, 1, 1, 1], and 2 more of the form [*q*_1_, *q*_2_, *q*_3_, *q*_4_, *q*_5_] where calculating *q*_*i*_ is done by setting *f*_*i*_(*q*_1_, *q*_2_, *q*_3_, *q*_4_, *q*_5_) = *q*_*i*_ and solving for *q*_*i*_. The probability of extinction is then given by $\Pi q_{i}^{i_{0}}$, where *i*_0_ is the initial number of infected individuals in category *q*_*i*_. Note that if *R*_0_ ≤ 1, then the extinction probability equals 1, and these equations for extinction probability only apply when *R*_0_ > 1.

$$\begin{array}{c} q_{1}=\frac{d_{C}}{d_{C}+\left(1-q_{3}\right)\alpha_{BC}\beta_{BC}} \end{array}$$ (13a)

$$\begin{array}{c} q_{2}=\frac{d_{M}}{d_{M}+\left(1-q_{3}\right)\alpha_{BM}\beta_{BM}} \end{array}$$ (13b)

$$\begin{array}{c} q_{3}=\frac{\alpha_{C}\beta_{BC}q_{1}q_{3}+\alpha_{M}\beta_{BM}q_{2}q_{3}+\left(\delta_{B}+d_{B}+\gamma_{B}\right)}{\alpha_{C}\beta_{BC}+\alpha_{M}\beta_{BM}+\left(\delta_{B}+d_{B}+\gamma_{B}\right)} \end{array}$$ (13c)

$$\begin{array}{c} q_{4}=q_{5}=1 \end{array}$$ (13d)

By substituting *q*_1_ and *q*_2_ into *q*_3_, we can find *q*_3_ which can be substituted back into *q*_1_ and *q*_2_. Now, the probability of extinction can be calculated as:

$$\begin{array}{c} P=q_{1}^{I_{C0}}\times q_{2}^{I_{M0}}\times q_{3}^{I_{B0}} \end{array}$$ (14)

### Parameter estimation

#### Data

We fit our model to the sentinel chicken seroconversion data of Florida, collected by Florida Department of Health [11, 41, 42]. Initial parameter ranges were defined by literature values when possible, as with mosquito biting rates [43], species composition [44] and host preference [29, 45]. For some parameters, we utilized the parameter ranges suggested in existing arboviral disease literature (e.g West Nile virus, Malaria, Zika, and Chikungunya) as proxies. For the parameters with no such prior knowledge, we used [0, 1] boundary as they are probabilities. Detailed information about the parameter ranges and related publications is given in S2 Appendix.

#### Model fitting and formulation

We solve a parameter estimation problem using the least square objective function as follows:

$$\begin{array}{cc} \underset{\vec{\theta }}{\text{Minimize}}\;\; & \sum_{w=0}^{W-1}{\left(\sum_{t=7w}^{7(w+1)}\frac{s(t,\vec{\theta })}{S_{B}\left(7w,\vec{\theta }\right)}-d\left(w\right)\right)}^{2} \end{array}$$ (15a)

$$\begin{array}{cc} \text{subject}\;\text{to}\; & s\left(t,\vec{\theta }\right)=\alpha_{BC}\beta_{BC}\frac{S_{B}\left(t,\vec{\theta }\right)}{N_{B}\left(t,\vec{\theta }\right)}I_{C}\left(t,\vec{\theta }\right)+\alpha_{BM}\beta_{BM}\frac{S_{B}\left(t,\vec{\theta }\right)}{N_{B}\left(t,\vec{\theta }\right)}I_{C}\left(t,\vec{\theta }\right) \end{array}$$ (15b)

$$\begin{array}{cc} & \frac{d}{dt}S_{C}\left(t,\vec{\theta }\right)=f_{1}\left(t,\vec{\theta },S_{C},I_{C},\cdots \right) \end{array}$$ (15c)

$$\begin{array}{cc} & \frac{d}{dt}I_{C}\left(t,\vec{\theta }\right)=f_{2}\left(t,\vec{\theta },S_{C},I_{C},\cdots \right) \end{array}$$ (15d)

$$\begin{array}{cc} & \;\;\;\;\vdots \end{array}$$

$$\begin{array}{cc} & S_{C}\left(t_{0}\right)=S_{C}\left(0\right),\;I_{C}\left(t_{0}\right)=I_{C}\left(0\right),\cdots \end{array}$$ (15e)

$$\begin{array}{cc} & \nu_{\text{lb}}\leq \int_{0}^{\text{T}}\left(N_{C}\left(t\right)+N_{M}\left(t\right)\right)dt/\left(\int_{0}^{\text{T}}N_{B}\left(0\right)dt\right)\leq \nu_{\text{ub}} \end{array}$$ (15f)

$$\begin{array}{cc} & \eta_{\text{lb}}\leq \int_{0}^{\text{T}}N_{C}\left(t\right)dt/\int_{0}^{\text{T}}\left(N_{C}\left(t\right)+N_{M}\left(t\right)\right)dt\leq \eta_{\text{ub}} \end{array}$$ (15g)

where the observed weekly seroconversion rates are given as *d*(*w*) for week *w*, *W* and *T* denote the number of weeks and days that we have in the seroconversion data, respectively. The formulation (15) minimizes the sum-of-squares error in the weekly seroconversion rates. The daily additional bird infection is calculated as $s(t,\vec{\theta })$ to express the weekly seroconversion rates in the simulation as (15b). Constraints (15c)–(15e) formulate the compartmental ODE system given in (1a)–(1k). The last two constraints (15f) and (15g) define the upper and lower bounds of the mosquito-to-bird ratio and the ratio of the enzootic vector out of all types of vectors, respectively. To find the parameter set ($\vec{\theta }$) that minimizes the squared error, we use sequential quadratic programming combined with basin-hopping method to solve the constrained minimization problem [46–48]. The minimization process is started at 10,000 different initial points extracted by the Latin hypercube sampling method. The bootstrapping approach for epidemic models suggested by Chowell is used to quantify the parameter uncertainty [49]. The results of uncertainty quantification can be found in both result section and S3 Appendix.

#### Parametrization of population ratios

It is known that the mosquito-to-bird population ratio or the relative population density is difficult to measure because one needs to observe the two populations at the same time. The mosquito density to bird values vary between existing studies [36, 50]. The upper and lower limits of the annual mosquito-to-bird ratio is set to 2 and 8 to align with existing literature [51, 52].

It is also difficult to measure the exact mosquito community composition in an area because mosquito habitats and feeding behaviors are significantly different by species and therefore counting results highly depends on the mosquito trap’s location and design [44]. The population ratio of the enzootic vector is assumed to be less than 10% (or *η*_ub_ = 0.1) because there exist multiple studies commonly reporting the portion of the mosquito species mostly feed on birds (*i.e. Culiseta melanura* and *Culex territans*) in the mosquito community is very small (smaller than 5%) [42, 44].

To satisfy the two population ratio constraints (15f) and (15g), the initial mosquito-to-bird population ratio ($\nu_{0}=\frac{S_{C0}+S_{M0}}{S_{B0}}$) and the initial enzootic-bridge vector population ratio (*η*_0_) are included as parameters. The following equations show how the two parameters *η*_0_ and *ν*_0_ determine the initial mosquito populations (*N*_*C*_(0) and *N*_*M*_(0)):

$$\begin{array}{cc} & N_{C}\left(0\right)=\eta_{0}\nu_{0}N_{B}\left(0\right) \end{array}$$ (16a)

$$\begin{array}{cc} & N_{M}\left(0\right)=\left(1-\eta_{0}\right)\nu_{0}N_{B}\left(0\right) \end{array}$$ (16b)

Because the disease is first reported before the first day of our observation data set [1], the initial population ratios for each disease compartment are also parameterized; four population parameters, $r_{I_{C}},r_{I_{M}},r_{I_{B}}$, and $r_{R_{B}}$ are added to determine the initial population of each compartment as follows:

$$S_{C}(0)=(1-r_{I_{C}}\;)N_{C}(0)$$ (17a)

$$I_{C}(0)=r_{I_{C}}N_{C}(0)$$ (17b)

$$S_{B}(0)=(1-r_{I_{B}}-r_{R_{B}})N_{B}(0)$$ (17c)

$$R_{B}(0)=r_{R_{B}}N_{B}(0)$$ (17d)

$$S_{M}(0)=(1-r_{I_{M}})N_{M}(0)$$ (17e)

$$I_{M}(0)=r_{I_{M}}N_{M}(0)$$ (17f)

$$I_{B}(0)=r_{I_{B}}N_{B}(0)$$ (17g)

## Results

In this section, we apply different analytic techniques to the deterministic and stochastic models with a parameter set that is fitted to the seroconversion data to diagnose the current state of EEE and analyze how it can potentially change. First, we compute ${\bar{R}}_{0}$ and the extinction probability to investigate the current impact of EEE. Then, we use the sensitivity analysis to reveal how much each parameter could potentially change the ${\bar{R}}_{0}$ of the EEE. At the end of this section, we explore three potential changes of EEE: (1) increased infectivity, (2) vertical transmission, and (3) changed host preference of the bridge vector. We examine these changes using what-if scenarios because it is not easy to inspect them in detail through sensitivity analysis.

### Simulation and result analysis

**Fig 3:**
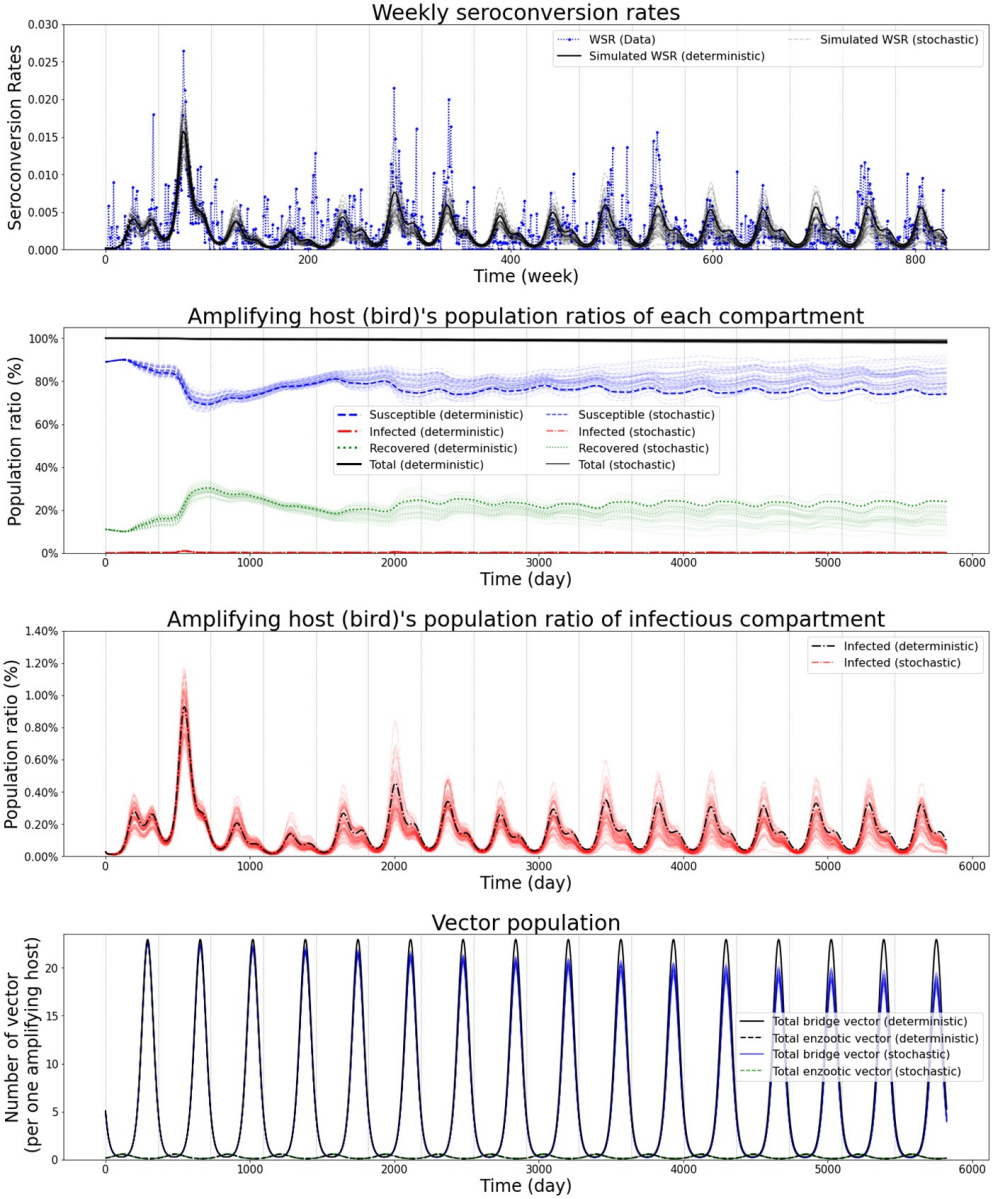
Simulation result. Simulation results of the deterministic model and stochastic model (50 instances). The 1st plot presents the weekly seroconversion data points (blue) and the simulated seroconversion rates in the two models. The remaining plots describe the population changes over time in the amplifying host compartment (2nd and 3rd) and the vector compartments (4th).

First, the deterministic model with the optimized parameter values is simulated by solving the corresponding ODEs to visually monitor the infectious population changing trend regarding EEE in different compartments. The details of the used parameter set that can be found in S2 Appendix, and the empirical distributions of each parameter and their confidence intervals can be found in S3 Appendix. We also simulate the stochastic model using the tau-leaping algorithm with the time step (*τ*) equal to 0.1 days, which is a commonly used value for *τ* in epidemiological simulations [53]. for 1,000 instances with the same parameter set to understand the stochastic outcomes of our model and to estimate the uncertainty of the parameter set by using the bootstrapping method [53]. It is noteworthy that the deterministic parameterization would lead to an underestimation of the basic reproductive ratio in the stochastic model according to Keeling and Rohani. For each time interval, the number of times each event in Table 1 occurs is taken as a number sampled from a Poisson distribution with the mean equal to *τ* multiplied by the transition rate for the event. The variables are updated to reflect the number of events that occurred, and this process is repeated until the end of the simulated time. To have stable simulation results for stochastic models, we set the initial population of the amplifying host at 400, 000 so that the community size is large enough following the suggestive result of Keeling and Grenfell’s study [54]. The simulation results are shown in Fig 3.

#### ${\bar{R}}_{0}$ and sensitivity analysis

**Fig 4:**
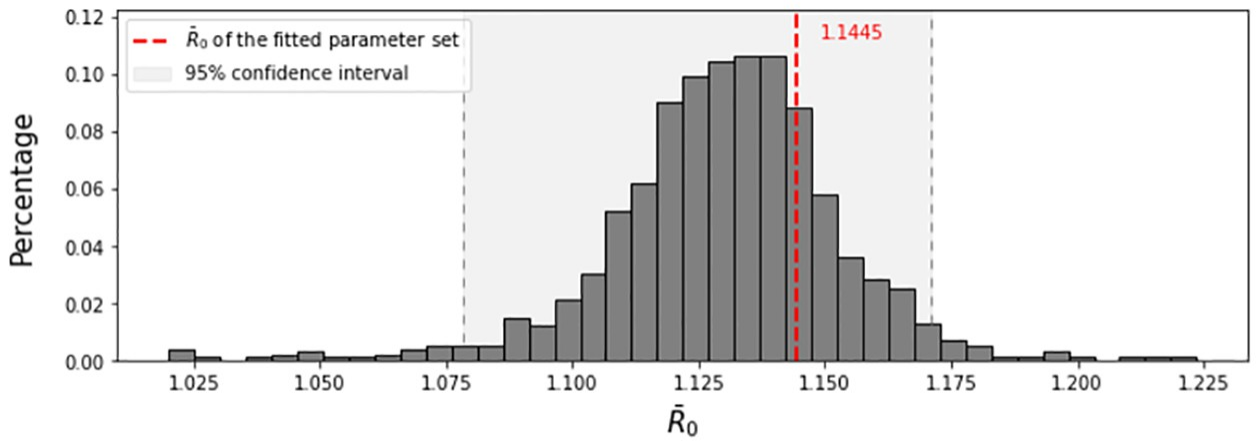
Empirical distribution of ${\bar{R}}_{0}$ obtained by the bootstrapping method. Fitted ${\bar{R}}_{0}$ is shown by the red dashed line (1.1445), and its 95% confidence interval is the grey region.

The fitted deterministic model has ${\bar{R}}_{0}=1.1445$, and Fig 4 shows the empirical distribution of ${\bar{R}}_{0}$ computed by the bootstrapping method, where the 95% confidence interval of ${\bar{R}}_{0}$ is [1.0783, 1.1711]. As expected from the long-lasting existence of EEEV in the USA, the ${\bar{R}}_{0}$ value is greater than 1.

**Fig 5:**
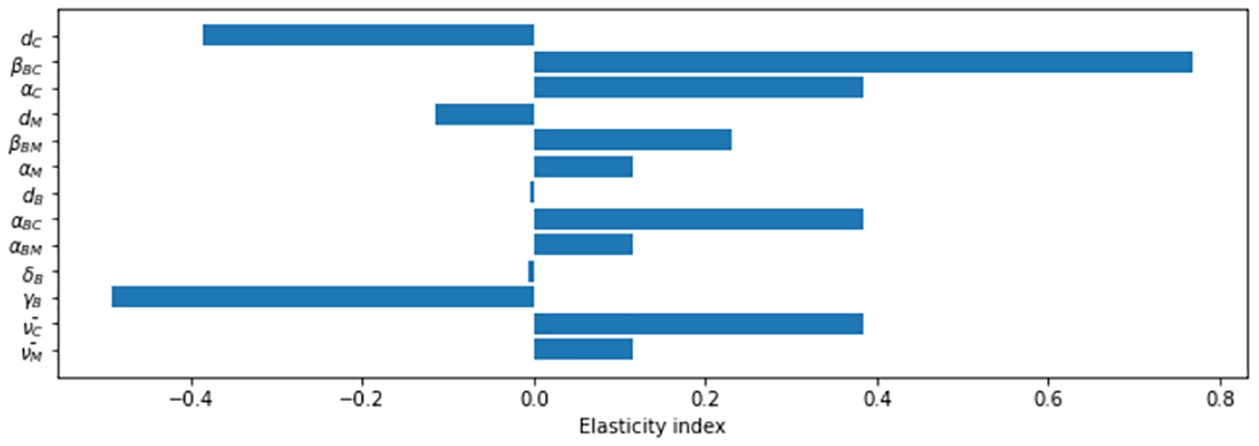
Sensitivity analysis results. X-axis corresponds to the elasticity index for the parameter. The magnitude of these values shows the strength of the relationship, while the sign tells whether the relative change in the parameter increases or decreases the magnitude of ${\bar{R}}_{0}$. For example, the elasticity index of *β*_*BC*_ is a large positive value meaning that an increase in the bird biting rate of the enzootic vector will result in a large increase in ${\bar{R}}_{0}$. On the other hand, *γ*_*B*_ has a large negative elasticity index meaning an increase in the bird recovery rate will decrease ${\bar{R}}_{0}$. Variables with elasticity values that are close to zero have little to no impact on ${\bar{R}}_{0}$ when they change.

${\bar{R}}_{0}$ ’s normalized sensitivities, or elasticity indices to each of the parameters are also calculated. The sensitivity values shown in Fig 5 represent the ratio of the relative change in ${\bar{R}}_{0}$ to the relative change in each parameter. In general, variables that are related to the enzootic vector compartment ($d_{C},\beta_{BC},\alpha_{C},\alpha_{BC},{\bar{\nu }}_{C}$) are 3.34 times more sensitive compared to their counterparts in the bridge vector ($d_{M},\beta_{BM},\alpha_{M},\alpha_{BM},{\bar{\nu }}_{M}$). This result is notable because the average population ratio between enzootic and bridge mosquitoes in the fitted model is about 4:96. So, even with a much smaller population size, the enzootic vector characteristics have a large impact on transmission. The result reconfirms that the enzootic vector plays key role in circulating EEE, while bridge vector has a greater role in causing damage to dead-end hosts. The recovery rate of infected birds (*γ*_*B*_) has the 2nd largest elasticity index magnitude among all parameters tested, which shows the importance of amplifying host’s infection duration in EEE circulation.

#### Extinction probabilities

Considering that EEE is an emerging virus with a limited geographical presence, the introduction of limited number of cases into a susceptible population is of concern. The success or extinction of these transmissions can be quantified through extinction probabilities. Using the parameters as solved in the previous section and the extinction probability function (14), extinction probabilities can be calculated for various initial conditions as shown in Table 2. Note that there are two solutions to *q*_3_, but one of the solutions produced negative and above one probability, which does not have a physical meaning and is ignored.

**Table 2:** Extinction probabilities for various initial conditions using parameters fitted to deterministic model. Columns represent the initial number of infections in enzootic vector (*I*_*C*0_), bridge vector (*I*_*M*0_), and bird (*I*_*B*0_) populations and the corresponding probability of extinction in the population.

*I*_*C*0_	*I*_*M*0_	*I*_*B*0_	Probability of extinction
1	0	0	0.4302
0	1	0	0.4496
0	0	1	0.3145
1	1	0	0.1934
1	0	1	0.1353
0	1	1	0.1414
1	1	1	0.0608

One case in the bridge vector population results in the highest probability of extinction at 44.96%. One case in the enzootic vector population results in a 43.02% probability of extinction, 4.31% lower than one case in the bridge vector. This difference is expected because, while bridge vectors are more meaningful for human cases, the survival of EEE is dependent on natural ecological reservoirs. Surprisingly, the probability of extinction found with one case in the bird population was 31.45%, the lowest probability of a single introduced infection. This could be due to the longer lifespan of birds or the fact that they are able to produce multiple types of infections that aren’t dead-end. This result is concerning due to the annual migration of many birds. Introducing cases to more than one compartment decreases the probability of extinction with a single case in all three compartments resulting in only a 6.08% probability of extinction.

### What-if scenarios: Hypothetical mutations and environmental changes

We also use the deterministic model that is fitted to the seroconversion data to explore hypothetical situations which are not easy to delve with sensitivity analysis. We evaluate the potential threats of EEE by simulating genetic mutation and environmental changes and measuring their impact.

Although EEEV mutations do not attract much attention due to its small number of cases, mutations can affect the spread of arboviral disease [55]. In fact, EEE is classified as a positive-strand RNA virus (or +ssRNA) according to Berman’s taxonomic guide to infectious diseases [56]. Peck and Lauring report that ssRNA viruses generally mutate faster than other groups of viruses [15], which is important as it may result in resistance, antibody escape, expanded host range, and other critical changes in disease dynamics. Global warming or environmental change is also accelerating the mutation of diseases and has a direct effect on the spread of diseases. For example, existing studies report that the infectivity of vectors can be increased by higher ambient temperature [57, 58].

To examine the impact of changes potentially caused by mutation and global warming, we establish the scenarios in this section and compare the result to the outcome we obtained from the default model. Among all the possible changes that can alter the parameter values described in our model, we test some of them selectively. Since our study is about the potential threat that EEE can cause, we create scenarios focusing on the change in the direction of increasing R0, as some existing studies in disease mutation suggested [59, 60].

The first scenario is increased infectivity, which can be caused by genetic mutation of EEE and increased ambient temperature. Second, we explore a change that extends the infectious period of EEE in bird hosts. Change in the duration of the infectious period is another scenario that is commonly tested in many other studies in epidemiology [61, 62]. To examine the impact, we decrease the *γ*_*B*_ value to simulate such mutation. In the third scenario, we simulate the situation where the bridge vector’s host preference is changed. The change can be caused by adaptive mutation, opportunistic behavior, or a change in mosquito species composition. According to existing studies, bridge vectors, or the mosquitoes that are infecting dead-end hosts such as horses, prefer mammal hosts compared to the avian hosts, or amplifying host of EEE [25, 27]. We change the feeding behavior of bridge vector with different host preference and assess the impact of changed host preference. Since our concern is the possible future outcomes, the final population ratio of the deterministic simulation in the result section is used as the initial population for the three what-if scenarios.

#### Increased infectivity

**Fig 6:**
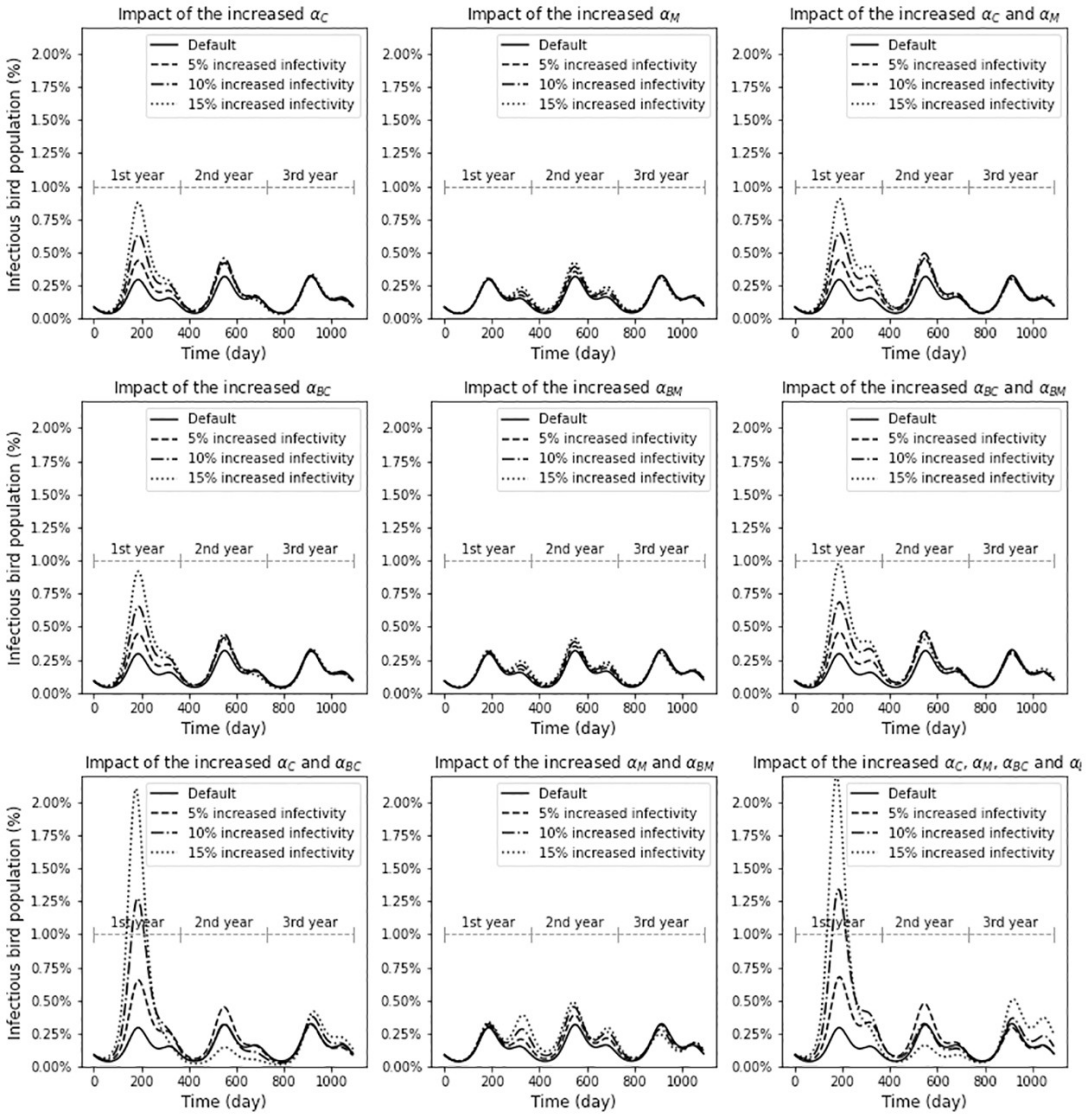
What-if scenarios with increased infectivity in different set of parameters. The bottom right shows the situation with all four infectivity parameters increased. In this case, 15% increased infectivity causes a large peak initially, which is subsequently dampened, but then elevates slightly again. In general, a greater increase in infections is seen with the increased infectivity in enzootic vectors than the change in bridge vectors.

Four different transmission rate parameters describe the infectivity of EEE: *α*_*C*_, *α*_*M*_, *α*_*BC*_, and *α*_*BM*_. *α*_*C*_ and *α*_*BC*_ represent the transmission rates of EEE from/to enzootic vectors while *α*_*M*_ and *α*_*BM*_ represent the transmission rates from/to bridge vectors. Each row of Fig 6 shows the simulation results where the infectivity values are increased in only enzootic vectors, only bridge vectors, and both vector types, respectively. In each column, we display the results where only the transmission rate to vector, only the rate from vector, and both rates are increased, respectively. In each case, we increase the selected transmission rates by 5%, 10%, and 15% and compare the host infection trends to the default case.

Fig 6 shows how the number of the infected amplifying hosts are affected by each change in transmission rates. In general, the magnitude of the impact is greater when infectivity increases in enzootic vectors than in bridge vectors. When *α*_*M*_ and *α*_*BM*_ are increased by 15%, the maximum peak size in bird infection increases only 1.53 fold. Whereas the peak size increases 6.58 fold when *α*_*C*_ and *α*_*BC*_ are increased by 15%. Also, increased infectivity in enzootic vector boosts the peak size in the first year’s host infection compared to the default case but the impact size is smaller in the 2nd and 3rd year. Moreover, in the 2nd year, the peak size can be smaller than the default result when the increase in the peak size is too large in the first year and cause an increase in immunity of the bird population. For example, when comparing the case with 15% increase in all infectivity parameter which obviously shows the most drastic change, to the default scenario, there is a ratio of 7.45 in the peak of the first year, but a ratio of 0.51 in the second year.

#### Extended infectious period

**Fig 7:**
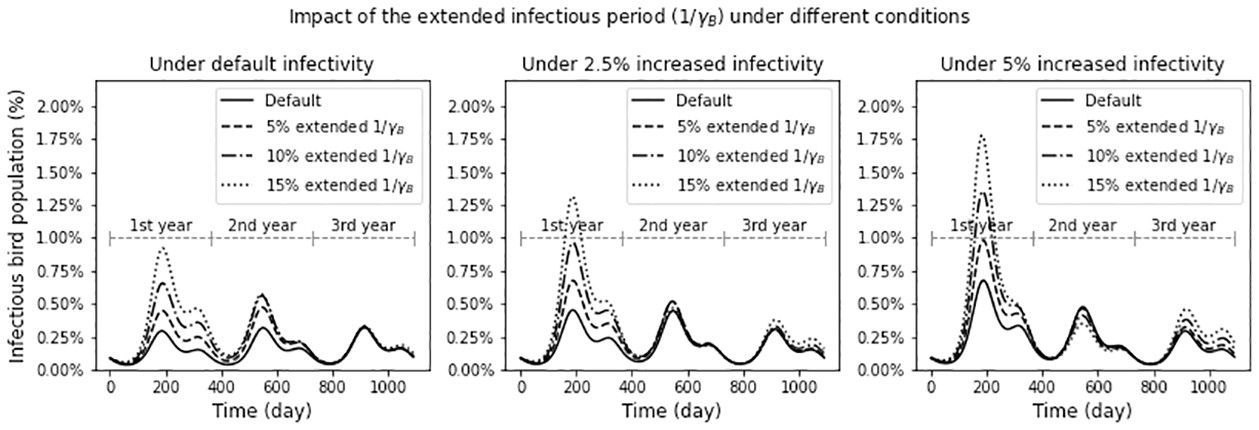
What-if scenarios with extended infectious period of EEE in bird hosts. The three plots show the trend in the infected bird population where the infectivity values (*α*_*C*_, *α*_*M*_, *α*_*BC*_, and *α*_*BM*_) are increased by 0% (default), 2.5%, and 5%, respectively. In each plot, trends that are changed by the increased infectious period by 5%, 10%, and 15% are presented.

The next scenario examines the impact of the extended infectious period of EEEV in bird hosts or reduced *γ*_*B*_. As we can see in the sensitivity analysis, decrease in the bird recovery rate will significantly increase the basic reproduction number. We explore how this type of mutation can have greater impact on EEE’s disease dynamics when it is combined with increased infectivity. Each plot in Fig 7 shows the simulation results where the infectivity values (*α*_*C*_, *α*_*M*_, *α*_*BC*_ and *α*_*BM*_) are increased by 0% (default), 2.5% and 5%, respectively. Under each infectivity condition, we increase the infectious period of EEE in hosts by 5%, 10%, and 15% and compare the trend of the infectious bird population to that of the default case.

Under the default infectivity condition (the left-most plot), the impact of the extended infectious period is similar to those of increased *α*_*C*_ or increased *α*_*B*_*C* shown in Fig 7 in the previous what-if scenario. The maximum peak increases 1.46, 2.02, and 2.83 fold when the infectious period is increased by 5%, 10%, and 15%, respectively. The increased infectivity amplifies the maximum peak even larger. When the infectivity parameters are increased by 2.5% and 5%, the peak size in the 1st year increases by 4.03 and 5.45 fold with a 15% extended infectious period compared to the default case. The result demonstrates that small changes in multiple parameters can result in a large impact on the disease dynamics of EEE. It also shows that similar disease dynamics can be driven by different combinations of changes in multiple parameters.

#### Change in host preference

It is known that bridge vector species of EEE prefer mammals more than birds, and only a small portion of bridge vector species has an opportunistic, evenly mixed feeding preference [27, 29, 30, 45]. In this what-if scenario, the bridge vector’s feeding behavior is changed to be more opportunistic and we compare the outcomes of this adjusted behavior to that of default scenario. That is, the proportion of *β*_*BM*_ in (*β*_*H*_ + *β*_*BM*_) is changed. As *β*_*BM*_ increases, the size of *I*_*M*_ increases, which also boosts the number of dead-end host infections because the number of vectors that biting hosts is increased. At the same time, *β*_*H*_ decreases, which negatively affects the number of dead-end host infections because now bridge vectors are less likely to bite dead-end host. Since all the other parameters are fixed, the value of the cumulative dead-end host infections in the next two-year period ($\beta_{H}\int_{0}^{T}I_{M}\left(t\right)dt$) is tracked to measure and compare the impact on the equine economy and public health. We also track the change in ${\bar{R}}_{0}$ to understand how the change in host preference affect EEE’s spread.

**Fig 8:**
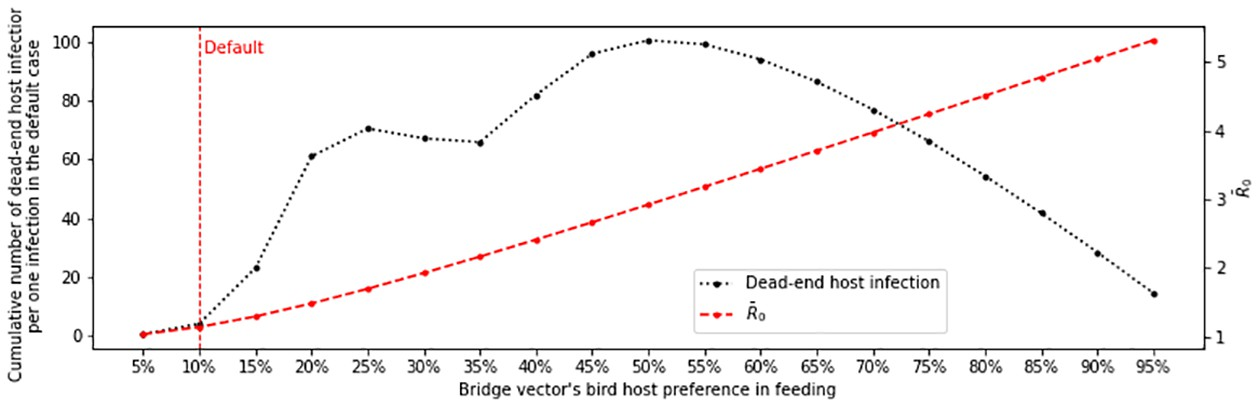
Cumulative dead-end host infections and ${\bar{R}}_{0}$ for different bridge vector host preference ratios. The current host preference is 10%. As feeding preference shifts from 90% dead-end host and 10% avian there is an increase in total dead-end host cases and ${\bar{R}}_{0}$. Disease burden is maximized at 50% dead-end host and 50% avian, while ${\bar{R}}_{0}$ is maximized at 0% dead-end host and 100% avian.

Fig 8 shows the result where values on the *x*-axis varies the bridge vector’s host preference ratio for birds (or $\frac{\beta_{BM}}{\beta_{BM}+\beta_{H}}$). The dead-end host infection size is maximized at 50% and increases significantly in the 10%-20% interval. Since the current default is known to be smaller than 10%, this result suggests that disease control authorities need to be wary of the influx of invasive mosquito species with opportunistic feeding behavior.

## Discussion

As a result of the 2019 spike in EEE cases, a greater understanding of the transmission patterns of EEE is needed. In this study, the multi-season transmission of EEE was explored using epidemic modeling methods. The modified basic reproductive number is greater than 1.0, which indicates that EEEV does not die out naturally in the current situation. Both the sensitivity analysis and the calculated extinction probabilities show the importance of the enzootic vector population size and properties in maintaining EEE transmission.

Using the epidemic model, potential viral and host mutations were also investigated by comparing the expected impact of each change. Both the increased infectivity and the extended infectious period result in greater annual peak size in the host infection, while the effect size of the increased infectivity is greater within the tested ranges. A change in mosquito biting behavior shows a peak in the dead-end host cases at 30 percent bird bites to 70 percent dead-end host bites. Further increases in bird biting reduce the dead-end host disease burden as the decrease in the dead-end host. This result indicates that the influx of mosquito species with rather more opportunistic feeding behavior than major species such as *Aedes aegypti* and the change in existing species’ feeding behavior can be an amplifier of damage caused by EEE. It suggests that we need to pay attention to such changes that can be caused by global warming and other environmental changes. In the appendices, we present the results of two additional what-if scenarios: one with vertical transmission and the other with vector control policy. The former shows that the existence of a vertical transmission path also can increase the peak in host infection. The latter presents that immediate vector control is one way to minimize the potential spread of virulent mutation in EEEV.

There are, of course, limitations to the presented model. The data used for fitting is from sentinel flocks of chicken in Florida [3, 41], so while available for every week it might not represent the full variation of different bird species [9]. Existing studies of West Nile Virus point out that the seroprevalence of an arbovirus in wild birds is significantly different depending on bird species, location, and age (adult or juvenile) [63–65]. Levine et al. report that blue jays (71.4%), northern cardinals (49.5%), and northern mockingbirds (52.3%) had significantly higher seroprevalence rates of WNV than other species such as American robins (15.3%) and Carolina wrens (10.6%) [64]. They also present that the highest probability of being seropositive for WNV is observed in the park areas with artificial water features for all wild bird species. Maquart et al.’s study of WNV on domestic birds also demonstrates that there are significant differences in seroprevalence rates for different species [65]. They report that a significantly higher seroprevalence was observed with geese and turkeys compared to that of chicken and ducks. For example, the seroprevalence of WNV in turkeys is greater than double that in chickens. Beveroth et al. present that there also exists a significant difference in the seroprevalence between adult (12.1%) and juvenile (5.5%) birds in Illinois [63]. Although these studies are about the seroprevalence of West Nile virus not EEE, we assume that the results of the study on WNV, one of the major arboviruses, are meaningful as a proxy given the lack of studies on the seroprevalence of EEE in wild birds. That is, it is possible that the dataset from sentinel chickens underestimates or overestimates the seroconversion rate of EEE for wild birds. There also exist studies of EEE suggesting that the dataset from sentinel chickens is potentially inaccurate. One study suggests that wilds birds within swamp areas lead to almost 2 times more EEE isolations than those outside of swamp areas [66]. Another study shows that 65% of sentinel chickens had EEE antibodies compared to 33% of wild birds nearby [67]. These suggest species and locations are also important for EEE data, emphasizing the parameter uncertainty associated with using the sentinel chicken data for calibrating the model. The species composition of the mosquito community also has great uncertainty because it highly depends on the environment surrounding the observation tools [44].

Additionally, assumptions were made for the model. Birds, enzootic vectors, humans, and bridge vectors were all assumed to be homogeneous populations without any variation in parameters or behavior. No spatial features were used. These are major simplifications that were selected due to the lack of data and can be adjusted in future works. Future work could address these issues by creating larger models and simulations.

Another assumption was that the only seasonal change was that of mosquito birth rates. There are multiple other potential seasonal factors. For example, people, mosquitoes, and birds are less active during the winter season resulting in lower biting rates. All the other seasonal factors, such as changes in human behavior, bird behavior, and bird migration, were ignored to keep the model as simple as possible. Due to these assumptions, detail is lost regarding any seasonal differences not arising from mosquitoes. Hypotheses about human behavior, bird behavior, and bird migration are also unable to be incorporated or tested without changing the model structure. However, the model can be iterated to add more complexity to address these questions in the future studies.

Our next goal is to use this model to explore hypotheses about the reintroduction of EEE each season from a location with year-round circulation. Moving forward this model could also be used to explore questions about migration and overwintering, or more importantly develop policies and interventions to reduce the dangers of EEE in the future.

## Conclusions

In this paper, we have established the first multi-season mathematical model for EEE transmission by using seasonal forcing to understand EEE’s repeating prevalence and the corresponding potential threats. We have utilized a 16-year seroconversion data set from sentinel chickens in Florida. By taking advantage of the seroconversion rates, we have circumvented the limitations caused by the scarcity of data about wild birds’ infections. Our analysis has confirmed that the roles of the enzootic vectors and the bridge vectors in EEE transmission are significantly different. While the former is more involved in the EEE’s persistence, the latter determines the direct impact on public health and the equine industry. Our results also show many hypothetical mechanisms that could lead to increased case numbers including increased infectivity, vertical transmission, and changes in feeding preference of bridge vectors. While our scenarios are hypothetical, the US has already experienced an increase in EEE cases in 2019. In 2020, human EEEV neuroinvasive cases in the United States fell from 38 to 13, still above the average of 11 human cases [68]. This could mirror the biannual pattern seen in our what-if scenarios, something that can be explored as case numbers for 2021 become available. The US also faces ongoing climate change, which further increases the probability of disease variation. What should be done about EEE is still unclear, but we have developed a valuable framework that can be used to test hypotheses about spread, mutations, interventions, and prevention.

## Supporting information

S1 Appendix Compartmental model without seasonal forcing and its analytic results. (PDF)

S2 Appendix Parameter table. (PDF)

S3 Appendix Empirical distributions of parameters obtained by the bootstrapping method and their 95% confidence intervals. (PDF)

S4 Appendix Additional what-if scenario 1. Vertical transmission.
(PDF)

S5 Appendix Additional what-if scenario 2. Vector control (reduced vector population).
(PDF)

S1 Raw image (TIF)

S2 Raw image (TIF)

S3 Raw image (ZIP)

S4 Raw image (ZIP)
