# Supplementary material for: Multi-season transmission model of Eastern Equine Encephalitis
Source: PLoS One. 2022 Aug 17;17(8):e0272130. doi: 10.1371/journal.pone.0272130 (PMC9385034; doi:10.1371/journal.pone.0272130)
Supplement: S1 Appendix — (PDF) [file pone.0272130.s001.pdf]

S1 Appendix A. Compartmental model without seasonal forcing and its analytic results

A-1. Schematic

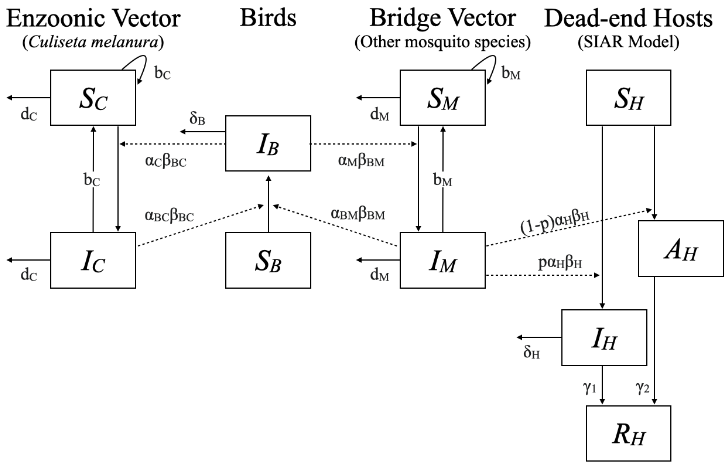

A-2. ODE system

$$\text{(Enzootic)}\begin{cases} \dfrac{d}{dt}S_C = b_C N_C - \alpha_C\beta_{BC}\dfrac{I_B}{N_B}S_C - d_C S_C & \text{(1a)} \\ \dfrac{d}{dt}I_C = \alpha_C\beta_{BC}\dfrac{I_B}{N_B}S_C - d_C I_C & \text{(1b)} \end{cases}$$

$$\text{(Bridge)}\begin{cases} \dfrac{d}{dt}S_M = b_M N_M - \alpha_M\beta_{BM}\dfrac{I_B}{N_B}S_M - d_M S_M & \text{(1c)} \\ \dfrac{d}{dt}I_M = \alpha_M\beta_{BM}\dfrac{I_B}{N_B}S_M - d_M I_M & \text{(1d)} \end{cases}$$

$$\text{(Birds)}\begin{cases} \dfrac{d}{dt}S_B = -\alpha_{BC}\beta_{BC}\dfrac{S_B}{N_B}I_C - \alpha_{BM}\beta_{BM}\dfrac{S_B}{N_B}I_M & \text{(1e)} \\ \dfrac{d}{dt}I_B = \alpha_{BC}\beta_{BC}\dfrac{S_B}{N_B}I_C + \alpha_{BM}\beta_{BM}\dfrac{S_B}{N_B}I_M - \delta_B I_B - \gamma_B I_B & \text{(1f)} \\ \dfrac{d}{dt}R_B = \gamma_B I_B & \text{(1g)} \end{cases}$$

$$\text{(Hosts)}\begin{cases} \dfrac{d}{dt}S_H = -\alpha_H\beta_H\dfrac{S_H}{N_H}I_M & \text{(1h)} \\ \dfrac{d}{dt}A_H = (1-p)\alpha_H\beta_H\dfrac{S_H}{N_H}I_M - \phi A_H - \gamma_2 A_H & \text{(1i)} \\ \dfrac{d}{dt}I_H = p\alpha_H\beta_H\dfrac{S_H}{N_H}I_M + \phi A_H - \gamma_1 I_H - \delta_H I_H & \text{(1j)} \\ \dfrac{d}{dt}R_H = \gamma_1 I_H + \gamma_2 A_H & \text{(1k)} \end{cases}$$

A-3. Basic reproduction number

$$R_0 = \sqrt{\frac{\alpha_C\beta_{BC}}{d_C}\frac{\alpha_{BC}\beta_{BC}}{\delta_B+\gamma_3}\frac{S_{C0}}{S_{B0}} + \frac{\alpha_M\beta_{BM}}{d_M}\frac{\alpha_{BM}\beta_{BM}}{\delta_B+\gamma_3}\frac{S_{M0}}{S_{B0}}} \quad (2)$$
