# Supplementary material for: Multi-season transmission model of Eastern Equine Encephalitis
Source: PLoS One. 2022 Aug 17;17(8):e0272130. doi: 10.1371/journal.pone.0272130 (PMC9385034; doi:10.1371/journal.pone.0272130)
Supplement: S2 Appendix — (PDF) [file pone.0272130.s002.pdf]

S2 Appendix B. Parameter tables. The table below shows the parameter values of the deterministic model fitted to the seroconversion data. We assign the ranges of parameters based on the given reference articles. We also obtained the 95% confidence interval of each parameter using the bootstrapping method with 1000 perturbed simulations. All values in the table are rounded to the fourth decimal place.

Parameter	Notation	Range	Fitted Value	Ref
1. Population ratio parameters				
Avg total-mosquito-to-bird population ratio	$\bar{\nu}$	$[2.00, 8.00]$	6.9377	[1,2]
Avg enzootic-vector-to-total-mosquito population ratio	$\bar{\eta}$	$[0.01, 0.50]$	0.0409	[3,4]
Infectious enzootic vector ratio at $t = 0$	$I_C(0)/N_C(0)$	$[0.00, 0.50]$	0.0000	
Infectious bridge vector ratio at $t = 0$	$I_M(0)/N_M(0)$	$[0.00, 0.50]$	0.0004	
Infected amplifying host ratio at $t = 0$	$I_B(0)/N_B(0)$	$[0.00, 0.50]$	0.0003	
Recovered amplifying ratio at $t = 0$	$R_B(0)/N_B(0)$	$[0.00, 0.50]$	0.1105	
2. Enzootic vector				
Natural birth & death rate	$b_C(= d_C)$	$[0.02, 0.07]$	0.0664	[5]
Probability of virus transmission to vector	α_C	$[0.25, 1.00]$	0.7740	[5]
Seasonal forcing (magnitude)	σ_C	$[0.20, 1.00]$	0.2260	
Seasonal forcing (peak location)	θ_C	$[-\pi/2, -\pi/6]$	-1.0693	[6]
3. Bridge vector				
Natural birth & death rate	$b_M(= d_M)$	$[0.02, 0.07]$	0.0595	[5]
Probability of virus transmission to vector	α_M	$[0.25, 1.00]$	0.9644	[5]
Seasonal forcing (magnitude)	σ_M	$[0.20, 1.00]$	0.6431	
Seasonal forcing (peak location)	θ_M	$[\pi/6, 5\pi/6]$	1.8950	[7]
4. Amplifying Host (Birds)				
Natural birth & death rate	$b_B(= d_B)$	Fixed	0.425 (per year)	[8]
Probability of transmission from enzootic vector	α_{BC}	$[0.25, 1.00]$	0.2918	[5]
Probability of transmission from bridge vector	α_{BM}	$[0.25, 1.00]$	0.2683	[5]
Death rate from virus	δ_B	$[0.00, 0.01]$	0.0021	[9,10]
Recovery rate from virus	γ_B	$[0.00, 0.33]$	0.1822	[10,11]

Table 1. Parameter table. Unless stated otherwise all rates are given in units of day^{-1}

References

1. Darbro JM, Harrington LC. Avian defensive behavior and blood-feeding success of the West Nile vector mosquito, Culex pipiens. Behavioral Ecology. 2007;18(4):750–757.

2. Hartemink N, Davis S, Reiter P, Hubálek Z, Heesterbeek J. Importance of bird-to-bird transmission for the establishment of West Nile virus. Vector-Borne and Zoonotic Diseases. 2007;7(4):575–584.

3. Giordano BV, Bartlett SK, Falcon DA, Lucas RP, Tressler MJ, Campbell LP. Mosquito Community Composition, Seasonal Distributions, and Trap Bias in Northeastern Florida. Journal of Medical Entomology. 2020;.

4. Hribar LJ. Dataset for mosquito collections on Big Pine Key, Florida, USA. Data in brief. 2019;26:104516.

5. Wonham M, Lewis M. A comparative analysis of models for West Nile virus. In: Mathematical epidemiology. Springer; 2008. p. 365–390.

6. Blosser EM, Lord CC, Stenn T, Acevedo C, Hassan HK, Reeves LE, et al. Environmental drivers of seasonal patterns of host utilization by Culiseta melanura (Diptera: Culicidae) in Florida. Journal of medical entomology. 2017;54(5):1365–1374.

7. Wilke AB, Vasquez C, Medina J, Carvajal A, Petrie W, Beier JC. Community composition and year-round abundance of vector species of mosquitoes make Miami-Dade County, Florida a receptive gateway for arbovirus entry to the United States. Scientific reports. 2019;9(1):1–10.

8. Wallace GJ. An introduction to ornithology [by] George J. Wallace [and] Harold D. Mahan. Third edition. ed. New York: Macmillan; 1975.

9. Stamm DD. Relationships of birds and arboviruses. The Auk. 1966;83(1):84–97.

10. Guy JS, Barnes HJ, Smith L. Experimental infection of young broiler chickens with eastern equine encephalitis virus and Highlands J virus. Avian diseases. 1994; p. 572–582.

11. Komar N, Dohm DJ, Turell MJ, Spielman A. Eastern equine encephalitis virus in birds: relative competence of European starlings (Sturnus vulgaris). The American journal of tropical medicine and hygiene. 1999;60(3):387–391.
