# Supplementary material for: Multi-season transmission model of Eastern Equine Encephalitis
Source: PLoS One. 2022 Aug 17;17(8):e0272130. doi: 10.1371/journal.pone.0272130 (PMC9385034; doi:10.1371/journal.pone.0272130)

S3 Appendix C. Empirical distributions of parameters obtained by the bootstrapping method and their 95% confidence intervals.

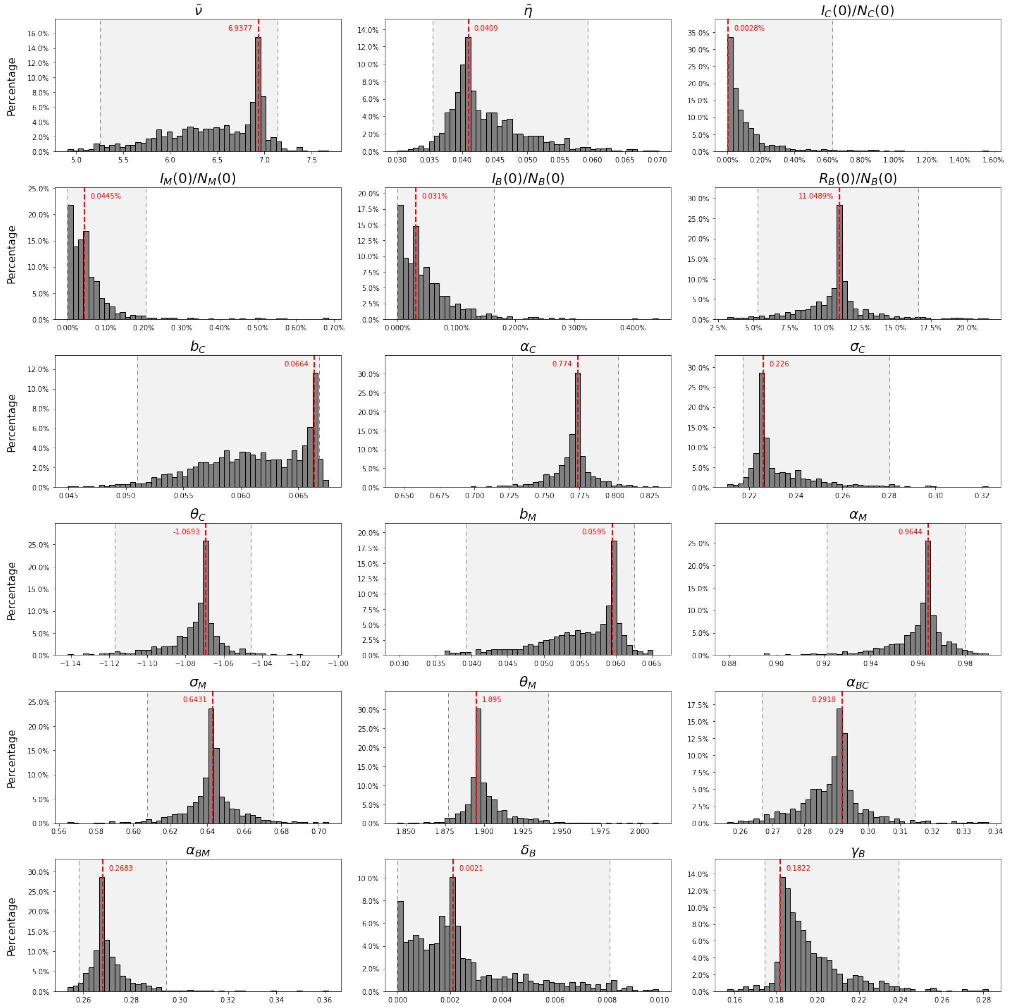

Supplement: S3 Appendix — (PDF) [file pone.0272130.s003.pdf]
