# Supplementary material for: Multi-season transmission model of Eastern Equine Encephalitis
Source: PLoS One. 2022 Aug 17;17(8):e0272130. doi: 10.1371/journal.pone.0272130 (PMC9385034; doi:10.1371/journal.pone.0272130)
Supplement: S4 Appendix — Vertical transmission. (PDF) [file pone.0272130.s004.pdf]

S4 Appendix D. Additional what-if scenario 1. vertical transmission. In this additional scenario, we explore a mutation that enables vertical transmission (passage of a disease-causing agent from mother to baby during the period immediately before and after birth). Note that the likelihood of a virus acquiring a different route of transmission has not been observed in prior arbor viruses and it is known that EEE is not vertically transmitted in any vector species [1]. That is, the plausibility of this scenario is low. We test this scenario to provide some insight into the disease dynamics of an EEE-like arbo virus with vertical transmission that may exist in the future. Most arboviruses are mainly transmitted horizontally among hosts and vectors, but still can be transmitted vertically from an infected female vector to its offspring [2].

To assess this scenario, a new recursive transition path is added from the infected vector compartment to the same compartment according to the schematic shown in Figure (1), and the host infection trends is examined by setting the vertical transmission rate to 10%, 20%, and 30%. The maximum vertical transmission rate of 30% is chosen based on the observed vertical transmission rates of other arboviruses, California encephalitis virus (CEV, 16%) and LaCrosse virus (LACV, 28%) [2].

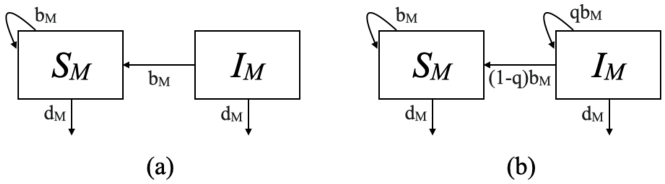

Fig 1. Schematic of birth and death transitions in bridge vector compartments (a) without vertical transmission and (b) with vertical transmission. A certain proportion (q) of newly born mosquitoes from the infected is born infected.

The amplifying host infection trends of the three cases are compared to that of the default model. First, we can observe that the change in trend is small when the vertical transmission is only added to the bridge vector compartment. When adding 30% vertical transmission in the enzootic vectors the peak size increases 6.19 fold in the first year and stays almost the same in the second year, but adding 30% vertical transmission to the bridge vector only yields 1.42 and 1.56 fold increases in the first and second years, respectively. This change is somewhat similar to the result that is observed in the 1st what-if scenario (Figure (**??**)) in that the change in enzootic vector compartment is more important compared to the change in bridge vector.

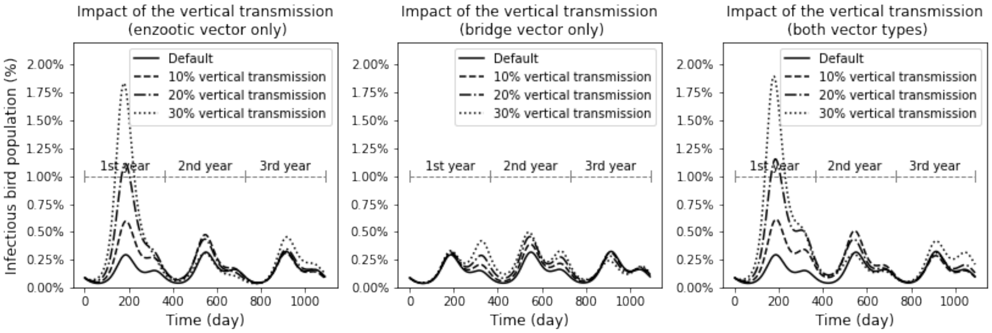

Fig 2. Number of the infected amplifying hosts in default cases and what-if scenario that introduces vertical transmission. The vertical transmission of the enzootic vector and bridge vector are explored separately and together.

References

1. Watts DM, Clark GG, Crabbs C, Rossi CA, Olin TR, Bailey CL. Ecological evidence against vertical transmission of eastern equine encephalitis virus by mosquitoes (Diptera: Culicidae) on the Delmarva Peninsula, USA. Journal of medical entomology. 1987;24(1):91–98.

2. Lequime S, Paul RE, Lambrechts L. Determinants of arbovirus vertical transmission in mosquitoes. PLoS pathogens. 2016;12(5):e1005548.
