# Supplementary material for: Multi-season transmission model of Eastern Equine Encephalitis
Source: PLoS One. 2022 Aug 17;17(8):e0272130. doi: 10.1371/journal.pone.0272130 (PMC9385034; doi:10.1371/journal.pone.0272130)
Supplement: S5 Appendix — Vector control (reduced vector population). (PDF) [file pone.0272130.s005.pdf]

S5 Appendix E. Additional what-if scenario 2. vector control (reduced vector population).

In this section, we examine how much vector population a community needs to eradicate to suppress the level of disease dynamics under that of the default case under different conditions. We assume that the goal of the vector control policy is to reduce the cumulative infection in birds in the next three years lower than the default case. The required vector control is negligibly small if there is no change in the duration of the infectious period ($1/\gamma_B$). However, it grows almost linearly if the γ_B stays the same and each transmission rate (α_C, α_M, α_{BC}, and α_{BM}) increases by 7%. In Fig (2), we can observe that the trend of linear increase starts from smaller increases in the transmission rates.

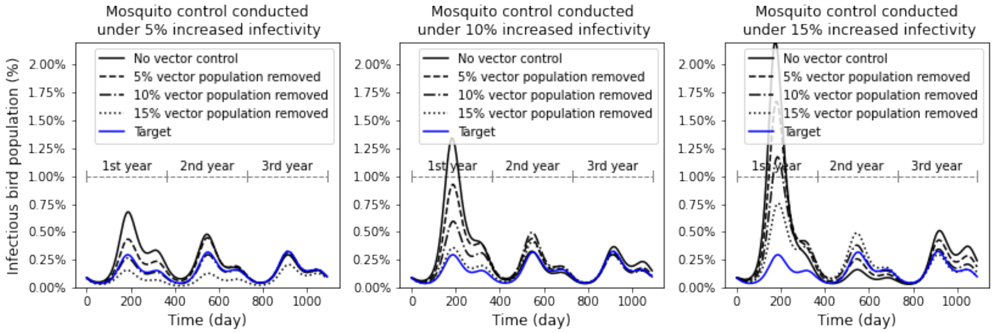

Fig 1. Number of the infected amplifying hosts in default cases and what-if scenario with changed infectivity parameter values and vector control. The blue lines in each plot represents the trend of default scenario or the target of vector control.

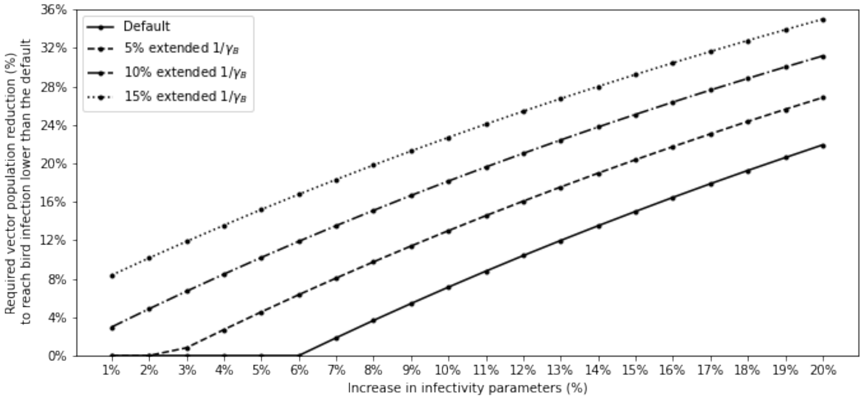

Fig 2. Required vector population reduction under different conditions The plot shows that the required vector control is negligibly small until parameters are changed to a certain extent but linearly increases after the threshold.
