# Supplementary figures and images for: Multi-season transmission model of Eastern Equine Encephalitis
Source: PLoS One. 2022 Aug 17;17(8):e0272130. doi: 10.1371/journal.pone.0272130 (PMC9385034; doi:10.1371/journal.pone.0272130)

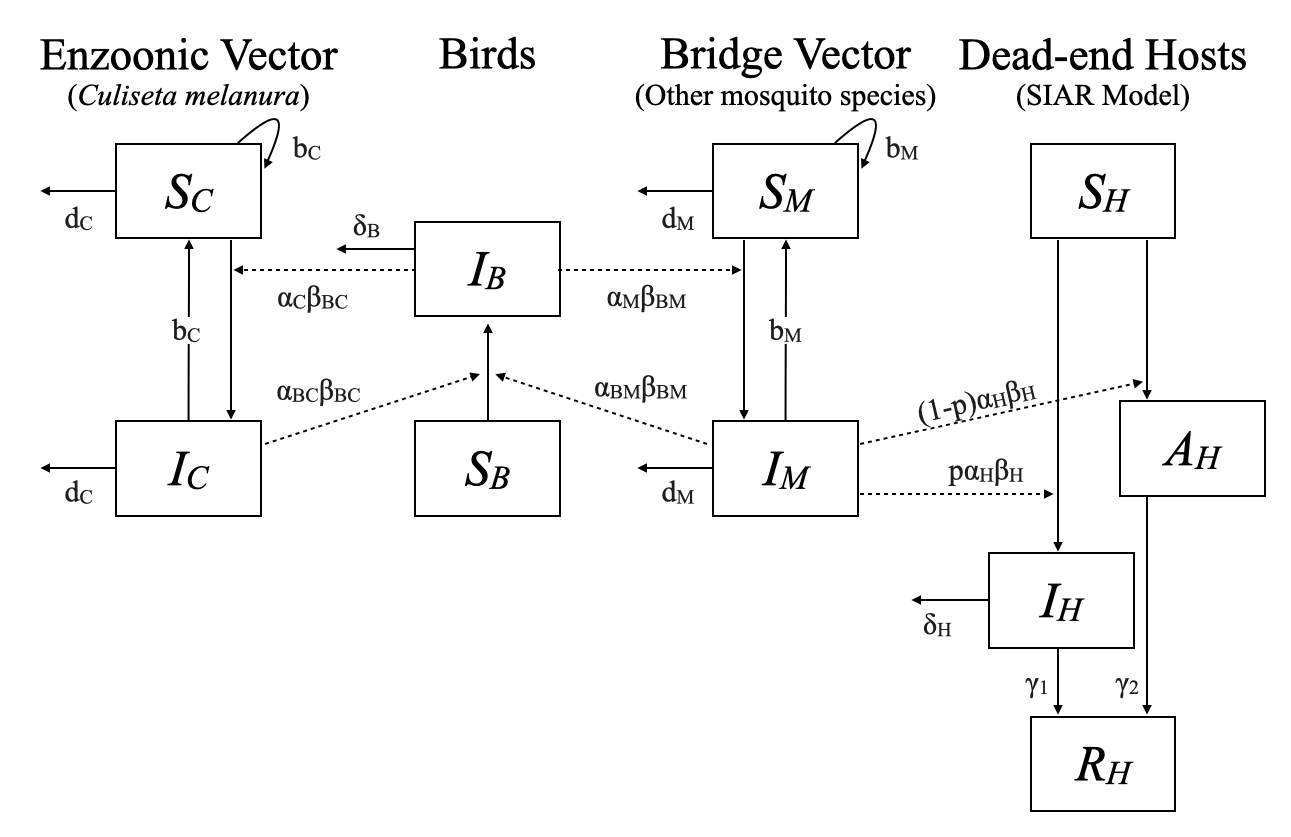

Supplement: S1 Raw image — (TIF) [file pone.0272130.s006.tif]

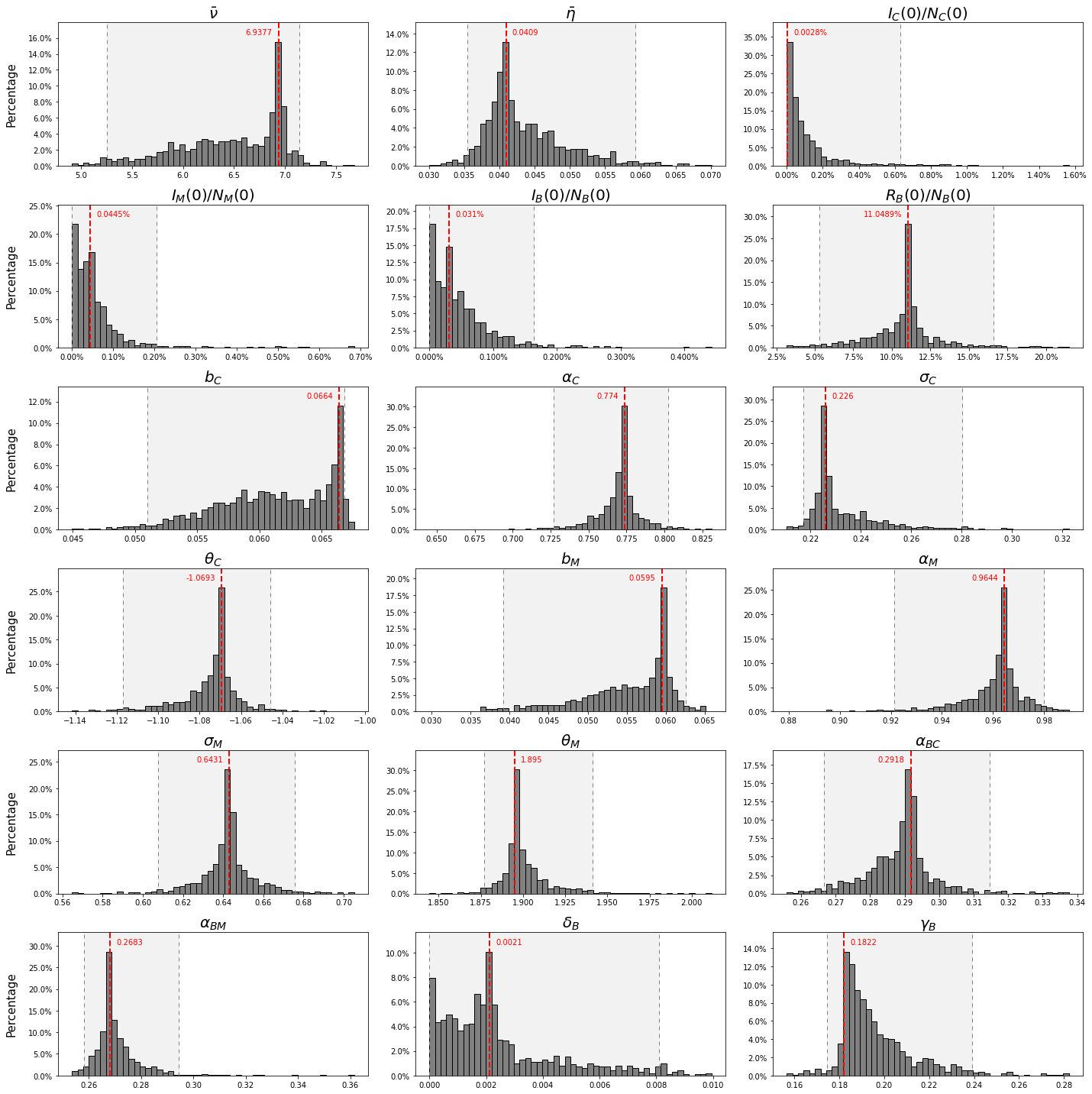

Supplement: S2 Raw image — (TIF) [file pone.0272130.s007.tif]

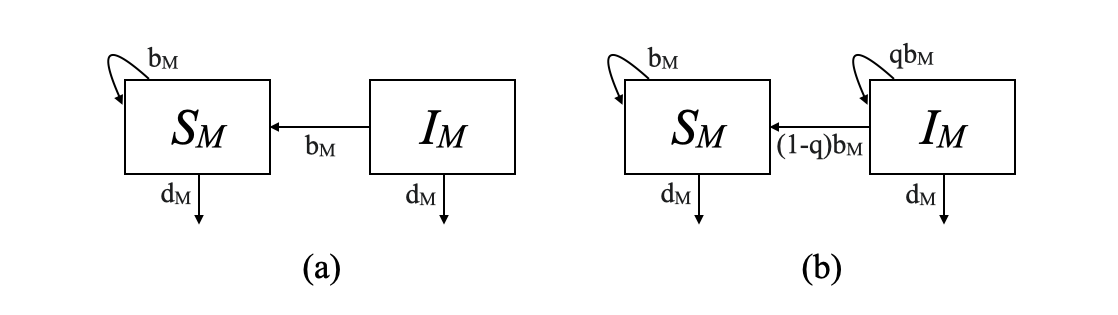

Supplement: S3 Raw image — (ZIP) [file pone.0272130.s008.zip › S3_raw_image_Fig1.tif]

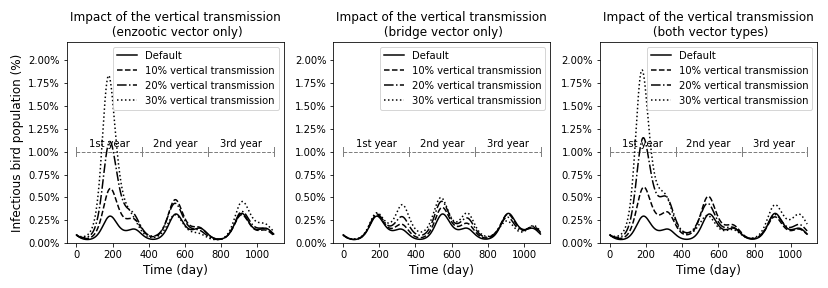

Supplement: S3 Raw image — (ZIP) [file pone.0272130.s008.zip › S3_raw_image_Fig2.tif]

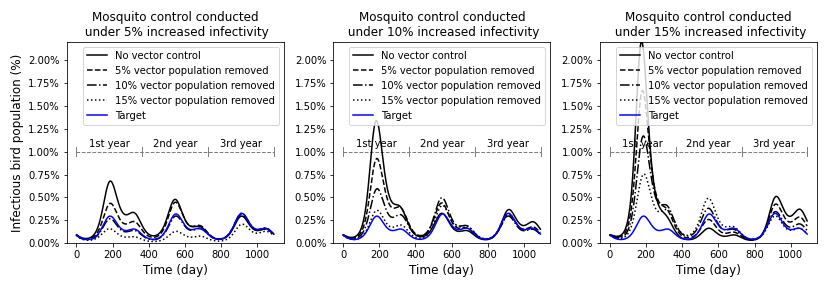

Supplement: S4 Raw image — (ZIP) [file pone.0272130.s009.zip › S4_raw_image_Fig1.tif]

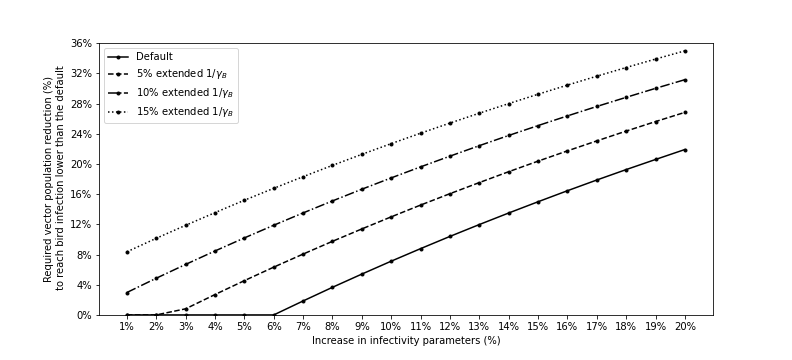

Supplement: S4 Raw image — (ZIP) [file pone.0272130.s009.zip › S4_raw_image_Fig2.tif]
